# Proline‐Rich Peptides with Improved Antimicrobial Activity against *E. coli*, *K. pneumoniae*, and *A. baumannii*

**DOI:** 10.1002/cmdc.201900465

**Published:** 2019-11-14

**Authors:** Mario Mardirossian, Riccardo Sola, Bertrand Beckert, Dominic W. P. Collis, Adriana Di Stasi, Federica Armas, Kai Hilpert, Daniel N. Wilson, Marco Scocchi

**Affiliations:** ^1^ Department of Life Sciences University of Trieste 34128 Trieste Italy; ^2^ Institute for Biochemistry and Molecular Biology University of Hamburg 20146 Hamburg Germany; ^3^ TiKa Diagnostics Ltd London SW17 0RE UK; ^4^ St Georges University of London London SW17 0RE UK

**Keywords:** Antibiotics, membrane permeabilization, proline-rich peptide, protein synthesis inhibition, solid-phase synthesis

## Abstract

Proline‐rich antimicrobial peptides (PrAMPs) are promising agents to combat multi‐drug resistant pathogens due to a high antimicrobial activity, yet low cytotoxicity. A library of derivatives of the PrAMP Bac5(1–17) was synthesized and screened to identify which residues are relevant for its activity. In this way, we discovered that two central motifs ‐PIRXP‐ cannot be modified, while residues at N‐ and C‐ termini tolerated some variations. We found five Bac5(1–17) derivatives bearing 1–5 substitutions, with an increased number of arginine and/or tryptophan residues, exhibiting improved antimicrobial activity and broader spectrum of activity while retaining low cytotoxicity toward eukaryotic cells. Transcription/translation and bacterial membrane permeabilization assays showed that these new derivatives still retained the ability to strongly inhibit bacterial protein synthesis, but also acquired permeabilizing activity to different degrees. These new Bac5(1–17) derivatives therefore show a dual mode of action which could hinder the selection of bacterial resistance against these molecules.

## Graphical abstract

**By screening a library** of derivatives of the proline‐rich antimicrobial peptide Bac5 (1–17), we identified the amino acids that are crucial for its antimicrobial activity. These findings allowed us to design new derivatives of Bac5 (1–17) with improved and wider activity spectrum against bacterial pathogens as well as maintaining a very low cytotoxicity. These new peptides possess a dual mechanism of action that should hinder the development of resistance against them.

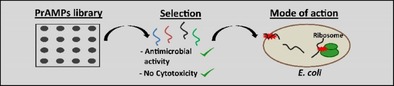

## Introduction

Multi‐drug resistant (MDR) pathogens are a concerning health problem worldwide that will seriously compromise many medical procedures, from routine to life‐saving treatments.^1^ Thus, the demand for novel antibacterial drugs with new mode of action is urgent. In this context, antimicrobial peptides (AMPs) have received considerable attention as potential lead compounds for the development of new antibiotics, however, they are often limited by cytotoxic activity and possible non‐specific or even undesired interactions within the host's body.^2^ By contrast, the subclass of proline‐rich antimicrobial peptides (PrAMPs) has potent antimicrobial activity against some of the most alarming bacterial species for MDR infections, such as *Klebsiella pneumoniae*, *Acinetobacter baumannii* and *Escherichia coli* 1, 3 and, at the same time, they are generally characterized by a low cytotoxic profile.^3^ One reason for this might be that, unlike many AMPs, PrAMPs do not kill bacteria by damaging their membranes,^4^ but rather enter the bacterial cytosol using primarily the inner membrane transporters SbmA, and to a minor extent the MdtM complex.^5^ Once inside the bacterial cell, many PrAMPs cause a lethal inhibition of protein synthesis by targeting mainly the ribosome.^6^ Derivatives of the mammalian PrAMPs Bac7 and Tur1 A, as well as of the insect PrAMPs oncocin, pyrrhocoricin, metalnikowin and apdidaecin, share overlapping binding sites located within the exit tunnel of the ribosome.^7^ These PrAMPs inhibit protein synthesis either by blocking the binding of the aminoacyl‐tRNA to the peptidyltransferase center during translation elongation,7c, 8 or by trapping the decoding release factors on the ribosome during the translation termination phase.7a, 9

Many studies reduced the length of PrAMPs to lower the cost of synthesis while maintaining their antimicrobial properties.^10^ Other studies have characterized fragments, derivatives or dendrimers of PrAMPs by often modifying their amino acid sequence by one or few residues. In addition, the rational design of new PrAMPs was reported (see for example^11^). Synthetic peptide arrays on cellulose support also known as SPOT synthesis (see^12^) allows the easy production of peptide libraries and was successfully applied to insect‐derived PrAMPs to broaden their spectrum of activity and improve their antimicrobial properties compared with their native molecules.^13^

Bac5 is a bovine proline‐rich cathelicidin, whose mode of action was recently described.6f Bac5 fragments inhibit bacterial protein synthesis6f and are mostly active against Gram‐negative pathogens,^14^ including *E. coli*, *A. baumannii* and *Salmonella enterica* ser. *typhimurium*.11b, 14 The N‐terminal 1–17 fragment of Bac5 retained antimicrobial activity and the same overall mechanism of action to inhibit protein synthesis as other PrAMPs.11b However, because of the lack of homology between Bac5 and other PrAMPs, as well as the absence of a structure of Bac5 on the ribosome, it has remained unclear which residues of Bac5 are critical for its translational inhibitory activity.

Here we have prepared three consecutive substitution libraries of Bac5(1‐17), a Bac5 fragment with a length compatible with the SPOT‐synthesis. The Bac5(1‐17) peptide was systematically modified to identify key residues for antimicrobial activity. Moreover, by single and multiple residues substitutions, we identified peptides with improved antimicrobial activity and broader spectrum of action due to a moderate membrane permeabilizing effect.

## Results

### Identification of Bac5(1–17) Residues Essential for Antimicrobial Activity

**Figure 1:**
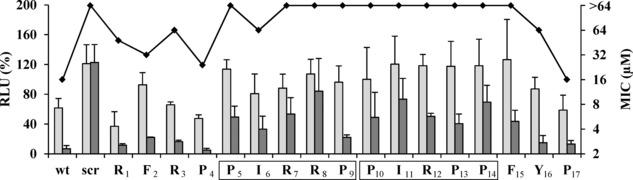
***In vitro*** **transcription/translation and MIC assays monitoring inhibitory activity of Bac5(1–17) and its derivatives**. Transcription/translation reactions (bar graph) were performed in presence of 10 μM (light gray) and 100 μM (dark gray) peptide. The luminescence (RLU%) of the translated luciferase (left hand Y‐axis) was normalized using the untreated controls and reported as average and standard deviation of at least three independent experiments. The peptide sequence is given on the X‐axis, and the values above each residue refer to the peptide carrying the alanine substitution in that position. The boxed sequences highlight the two PIRXP sequences. The minimal inhibitory concentration (MIC) for the Bac5(1–17) derivatives (line graph with black diamonds) is shown with tested concentrations indicated on right hand Y‐axis. Unmodified wild type (wt) and scrambled (scr) Bac5(1–17) peptides were used as controls for both MIC and transcription‐translation assays.

To identify key residues for the antimicrobial activity of Bac5(1–17), an alanine‐scan was performed. The seventeen Bac5(1–17) derivatives were tested for their capability to inhibit protein synthesis *in vitro* using coupled transcription/translation assays [6a, 6f, 7c, 8a, 8b, 11b] as well as to inhibit the growth of *E. coli* cells using minimal inhibitory concentration (MIC) assays in Müller‐Hinton broth (MHB) (Figure 1). Ten out of 17 alanine‐substituted Bac5(1–17) derivatives did not display any antimicrobial activity (MIC>64 μM). These included alanine‐substitutions in almost all the positions of the two repeats of the ‐PIRXP‐, whereas the N‐ and C‐terminal residues were more tolerant to substitutions as seen by an MIC comparable to the wild type Bac5(1–17). As expected, a scrambled Bac5(1–17) control peptide displayed no antimicrobial activity (MIC>64 μM) and did not inhibit protein synthesis *in vitro*. Generally, the trend of protein synthesis inhibition of the Bac5(1–17) derivatives paralleled the activity in the MIC assay (Figure 1), e. g. the wild type Bac5(1–17) exhibited 50 % inhibition at 10 μM and nearly complete inhibition at 100 μM, similarly to the N‐ and C‐terminal substituted Bac5(1–17) derivatives. By contrast, alanine‐substitutions in the PIRXP repeats generated peptides that had little if any inhibitory activity at 10 μM and required 100 μM to observe inhibition. These findings suggest that the core PIRXP repeats are critical for interaction of Bac5(1–17) with the ribosome.

### SPOT‐Synthesis Screen for Bac5(1–17) Derivatives with Improved Antimicrobial Activity

To identify Bac5(1–17) derivatives with improved activity, a SPOT‐synthesis was performed where further substitutions were introduced individually at each and every position of the Bac5(1–17) sequence. Specifically, we inserted residues with distinct chemical and physical characteristics, such as Gly (G, no side chain, small), Ser (S, small, polar), Arg (R, large, positively charged, hydrophilic), Pro (P, restricts conformation), Glu (E, medium, negatively charged, hydrophilic), Phe (F, large, aromatic, one ring, hydrophobic) and Trp (W, large, aromatic, two rings, hydrophobic) in every position of the Bac5(1–17) sequence, generating 122 derivatives. The MIC was then determined using *E. coli* BW25113 in MHB to assess whether the Bac5 derivatives had altered antimicrobial activities compared to the wild type and scrambled Bac5(1–17) peptide controls (Table 1). Similar to the alanine‐scanning, glycine substitutions within the core of the peptide lead to a loss of antimicrobial activity, whereas substitutions at the N‐ and C‐termini were better tolerated. By contrast, substitutions of Glu, Pro and Ser led to dramatic loss of the antimicrobial activity (MIC 64 μM or greater) regardless of the position, with the single exception of P4S that had an MIC (16 μM) comparable with the wild type. While substitutions with Phe, Trp and Arg were better tolerated, overall only 11 of the 122 derivatives actually displayed stronger antimicrobial activity than the wild type. These included P4 substitutions with Trp, Phe, Arg as well as Gly. In addition, Trp at positions 2, 6, 15, 16 and 17 as well as Arg at positions 9 and 17 led to improved MIC values. Thus, in total, substitutions at seven distinct positions (2, 4, 6, 9 and 15–17) led to MIC improvements. Interestingly, three of the seven positions were prolines (P4, P9 and P17), indicating that prolines at these positions are not crucial for the antimicrobial activity of the Bac5(1‐17) derivatives.

**Table 1:** MIC values (μM) of the native Bac5(1‐17), of its substitution variants and of a scrambled form against *E. coli* BW25113.

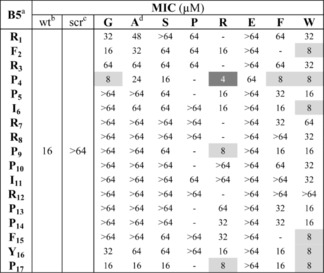

Grey shades highlight substitutions improving antimicrobial activity compared to the native Bac5(1‐17); ^[a]^ Bac5(1‐17) sequence and position numbers; ^[b]^ Bac5(1–17) wild‐type; ^[c]^ Bac5(1‐17) scrambled; ^[d]^ The Ala‐scan MIC values from Figure 1 are also included in this table for comparison. Results are the median of at least three independent experiments (n≥3). The scrambled peptide (scr) sequence was: FPIRYRPFRRPPRPIPP.

### Antimicrobial Activity and Cytotoxicity of Bac5(1–17) Multi‐Substituted Derivatives

The single amino acid substitutions improving the antimicrobial properties of Bac5(1‐17) were then combined to design new peptides (Bac5 peptides 265 to 296 in Table 2). This new peptide library was first screened for antibacterial activity against *E. coli* BW25113 and then assayed by a tetrazolium salt assay (MTT) for cytotoxicity toward the human lymphocytic leukaemia cell line MEC‐1. A two‐fold increase in MIC values was observed for some of the same peptides displayed in Table 1 (from 260 to 264) due to the intrinsic variability of peptide purity among the different SPOT‐syntheses. However, with the exception of the peptide 262, all of these derivatives were equally or more active than the wild type Bac5(1–17) with many of them displaying MIC values of 4 μM. Generally, the antimicrobial activity increased with the number of substitutions, such that the single substituted peptides displayed a median MIC of 16 μM, di‐ and tri‐substituted peptides 8 μM, and tetra‐substituted peptides 4 μM. Although three penta‐substituted peptides displayed a MIC of 4 μM (Bac5‐derivatives 291, 293, 294), additional substitutions often led to a worse MIC as seen when comparing 281 with 290 or 282 with 292 (MIC 4 increased to 8 μM) as well as 289 with 296 (MIC 8 increased to 16 μM). Next, the cytotoxicity of the peptide library was assessed by monitoring the viability of human lymphocytic leukaemia cells (MEC‐1) treated with 32 μM of each peptide (Table 2). Only two Bac5 peptides (273 and 293) displayed severe cytotoxicity, reducing the viability of treated cells by 32 % and 41 %, respectively.

**Table 2:** MIC (μM) values measured against *E. coli* BW25113 and effects of 32 μM of the second generation Bac5(1‐17) derivatives on MEC‐1 cell viability (Viab, % of untreated controls).

Code	Sequence	N°(+)	MIC	Viab%	Code	Sequence	N°(+)	MIC	Viab%
wt	RFRPPIRRPPIRPPFYP	+5	32	108	277	RWRRPIRRRPIRPPFYP	+7	8	99
**258**	**RFRPPIRRPPIRPPFYR**	**+6**	**8**	**95**	**278**	**RWRWPIRRPPIRPPFYR**	**+6**	**4**	**86**
259	RFRRPIRRRPIRPPFYP	+7	8	105	279	RFRWPIRRRPIRPPFYR	+7	8	84
260	RWRPPIRRPPIRPPFYP	+5	16	81	280	RFRWPIRRRPIRPPFYW	+6	4	78
261	RFRWPIRRPPIRPPFYP	+5	8	82	**281**	**RWRRPIRRRPIRPPFYW**	**+7**	**4**	**81**
262	RFRPPIRRPPIRPPWYP	+5	32	81	282	RWRRPIRRRPIRPPFYR	+8	4	99
263	RFRPPIRRPPIRPPFWP	+5	16	81	283	RWRRPIRRRPIRPPWYP	+7	4	91
264	RFRPPIRRPPIRPPFYW	+5	16	95	284	RFRWPIRRRPIRPPWYR	+6	4	72
265	RFRRPIRRPPIRPPFYR	+7	8	91	285	RFRRPIRRRPIRPPFWR	+8	8	90
266	RFRRPIRRRPIRPPFYR	+8	16	87	286	RFRWPIRRRPIRPPFWR	+7	4	80
267	RWRPPIRRPPIRPPWYP	+5	16	75	287	RFRRPIRRRPIRPPWYR	+8	8	95
268	RFRWPIRRPPIRPPWYP	+5	8	93	288	RRRRPIRRRPIRPPFYR	+9	16	102
269	RWRWPIRRPPIRPPFYP	+5	8	92	289	RRRRPIRRRPIRPPWYP	+8	8	95
270	RWRWPIRRPPIRPPWYP	+5	8	90	290	RWRRPIRRRPIRPPWYR	+8	8	91
271	RFRWPIRRRPIRPPFYP	+6	4	98	**291**	**RWRRPIRRRPIRPPFWR**	**+8**	**4**	**96**
**272**	**RFRWPIRRPPIRPPFYR**	**+6**	**4**	**102**	292	RWRRPWRRRPIRPPFYR	+8	8	78
273	RFRRPIRRPPIRPPWYP	+6	12	68	293	RWRWPIRRRPIRPPWYR	+7	4	59
274	RWRRPIRRPPIRPPFYP	+6	8	84	294	RWRWPIRRRPIRPPFWR	+7	4	73
275	RWRRPIRRPPIRPPFYR	+7	8	89	295	RRRRPWRRRPIRPPFYW	+8	8	77
276	RWRRPIRRPPIRPPWYP	+6	8	83	296	RRRRPIRRRPIRPPWYR	+9	16	95

Results of the MIC and cell viability assays are reported as the median or the average, respectively, of at least 3 independent experiments N° indicates the number of charges. The sequences of the peptides selected for synthesis on resin are in bold.

Five of the screened peptides were then further characterised. They were representative of a progressively increasing number of substitutions (from 1 to 5), displaying low MIC values and high biocompatibility towards eukaryotic cells (viability %).

The mono‐substituted peptide 258 was selected so as to include an intermediate substitution state linking the wild type Bac5(1‐17) and di‐substituted peptide 272 (containing P4W and P17R), which was the most promising di‐substitute derivative (Table 2). The peptide 278 was selected among the tri‐substituted derivatives because of its lower MIC value. Regarding the tetra‐substituted peptides, the 281 was preferred over 282 to evaluate the effects of a C‐terminal Trp. Lastly, the peptide 291 had the lowest MIC value and did not display any cytotoxicity.

Despite SPOT synthesis provides a powerful initial screening tool, the produced peptides are un‐purified. For this reason the five selected Bac5(1‐17) derivatives (258, 272, 279, 281 and 291), as well as the wild‐type Bac5(1–17), were singularly re‐synthesized by resin‐based synthesis at ≥95 % purity. For all the subsequent experiments, only highly purified peptides were used.

### Inhibition of *in vitro* Transcription/Translation by Five Selected Bac5 (1–17) Derivatives

**Figure 2:**
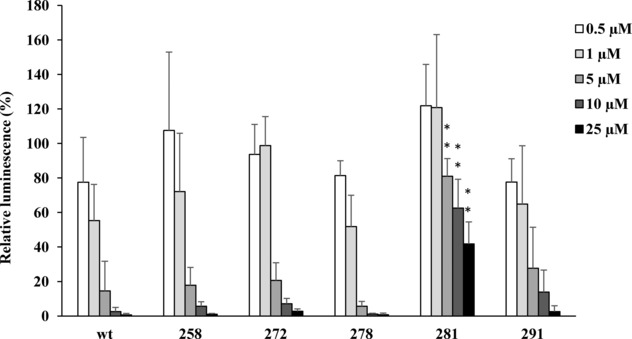
**Inhibition of** ***in vitro*** **transcription/translation by selected Bac5(1–17) derivatives**. Increasing concentrations of peptides were added to an *E. coli* lysate‐based transcription/translation reaction. Luminescence of the firefly luciferase reporter protein produced after 60 min is presented as relative luminescence compared to control samples, which lacked peptide and was normalized as 100 %. Error bars represent the standard deviation calculated on the average of at least 5 independent experiments (n=5). **=p‐value <0,01 versus the wt peptide (Student t‐test).

The ability of the five selected Bac5(1–17) derivatives (258, 272, 279, 281 and 291) to inhibit protein synthesis was then assessed using an *in vitro* transcription/translation assay based on *E. coli* lysates, and compared with that of the wild‐type peptide. Three of the derivatives (258, 272 and 278) displayed inhibitory activity comparable with that of the wild type Bac5(1‐17) peptide, with a near complete inhibition of translation occurring at 10 μM (see Figure 2). Importantly, since the antibacterial activities of these peptides were stronger than the wild type Bac5(1–17), this suggests that the improved MIC values arising from the amino acid substitutions is not due to an improved interaction with the ribosome. Indeed, this is emphasized by the 281 and 291 peptides which had superior MIC values (4 μM) and yet were respectively worse and slightly worse inhibitors than then wild type Bac5(1–17) in the transcription/translation assay (Figure 2). Collectively, these findings suggest that the improved antimicrobial activity of the substituted Bac5(1–17) derivatives compared to the wild type Bac5(1‐17) peptide (observed in Table 2) is not due to an improved ability to bind to ribosomes and inhibit translation (Figure 2).

### Membrane‐Permeabilizing Activity of Five Selected Bac5(1–17) Derivatives

**Figure 3:**
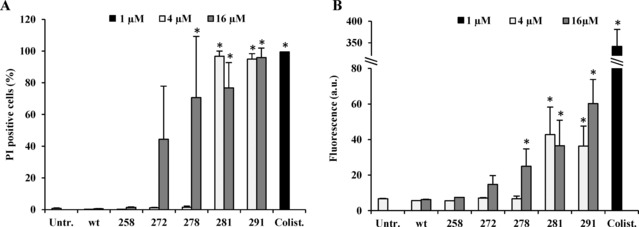
**Flow cytometric evaluation of** ***E. coli*** **BW25113 membranes permeabilization**. Propidium‐iodide uptake assay was performed after 30 min incubation in the presence of 4 μM and 16 μM of peptides. (A) The percentage of permeabilized cell in the bacterial population and (B) the average fluorescence of permeabilized bacteria were assessed. 1 μM of the lytic antibiotic colistin (Colist.) was used as a positive control of permeabilization. In the untreated control (Untr.) bacteria were treated with sterile water. Average and standard deviation of three independent experiments. *=p‐value <0.05 versus the wt peptide (Student t‐test).

The lack of an improved inhibitory activity of the Bac5(1–17) derivatives in the transcription/translation system indicates that their improved antibacterial activity is due to another mode of action. Given the number of positively charged arginine and tryptophan substitutions introduced into these peptides, an explanation for the improved activity could be that these peptides acquired a membrane‐permeabilizing ability. To test this hypothesis, the membrane integrity of *E. coli* cells was assessed in the presence of the selected peptides using flow cytometry measuring the fluorescence of propidium iodide (PI) uptake by the permeabilized bacterial cells. To evaluate how many bacteria were permeabilized by the peptides, the percentage of the bacterial population becoming positive to PI after exposure to the derivatives was calculated (Figure 3A). Afterwards, the extent of the membrane damage was estimated by measuring the intensity of fluorescence associated with the permeabilized cells (Figure 3B). As a control, the wild‐type Bac5(1–17) did not display any membrane damaging activity, which is in agreement with the literature and its known mode of action (Figure 3) [11b]. The Bac5 peptides 258, 272 and 278 displayed no permeabilizing effect at 4 μM, however, some membrane‐destabilizing activity was observed for 272 and 278 at higher (16 μM) concentrations (Figure 3). By contrast, 4 μM of the 281 or 291 peptides was sufficient to permeabilize 100 % of bacterial cells, to a similar extent as 1 μM colistin, the positive control used for permeabilizing activity [15]. However, it should be noted that even the highest fluorescence intensities measured by the most permeabilizing peptides at 16 μM were still only 20 % of that observed for bacteria treated with colistin used at 16‐fold lower concentrations (1 μM) (Figure 3B). These results show that the 272 and 278 peptides gained a moderate degree of membrane permeabilization activity, observable only at high concentrations, whereas the 281 and 291 peptides exerted permeabilization activity even at low (yet bactericidal) concentrations. By contrast, the wild type Bac5(1–17) and 258 peptide lacked any permeabilizing activity, even at high peptide concentrations. Collectively, these results suggest that improved antimicrobial activity of the 281 and 291 peptides compared to wild type Bac5(1–17) may result from their newly acquired ability to destabilize the bacterial membrane.

### Influence of SbmA and Evaluation of the Activity Spectrum of Bac5(1‐17) Derivatives

Since the membrane integrity assays indicated that the 281 and 291, and to a lesser extent 272 and 278 peptides, exhibited permeabilizing activity, whereas the 258 peptide did not, we were interested in assessing the influence of the SbmA transporter on the antimicrobial activity of these peptides. To do this, MIC assays were performed using the *E. coli* BW25113 parental strain as well as the BW25113ΔsbmA strain lacking this inner membrane transporter (Table 3). As expected, Bac5(1‐17) displayed antimicrobial activity (MIC 16 μM) against the parental strain, but not the BW25113ΔsbmA strain (MIC >64 μM), consistent with the essential role of SbmA for the internalization of this peptide [5a]. By contrast, the 272, 278, 281 and 291 peptides all displayed excellent activity against both the parental and Δ*sbmA* strain, with only a 2‐fold activity reduction being observed in the absence of the transporter (Table 3). This data indicates a mechanism of action that does not require the use of the SbmA transporter. The 258 peptide displayed antimicrobial activity (MIC 8 μM) against the parental strain, but was also ineffective against the Δ*sbmA* strain (MIC 64 μM), consistent with the lack of permeabilization activity (Figure 3).

**Table 3:** MIC and MBC of the native Bac5(1–17) (wt) and selected Bac5‐derivatives on pathogenic and reference bacterial strains.

	MIC (μM)						MBC (μM)					
Bacteria	wt	258	272	278	281	291	wt	258	272	278	281	291
*E. coli* BW25113	16	8	4	2	2	2	>64	64	8	4	2	8
*E. coli* BW25113Δ*sbmA*	>64	64	8	8	4	4	>64	>64	16	8	8	8
*K. pneumoniae* ATCC 700603	>64	>64	24	16	64	4	>64	>64	>64	48	>64	>64
*A. baumannii* ATCC 19606	>64	64	8	4	2	8	>64	64	16	8	8	16
*P. aeruginosa* ATCC 27853	>64	>64	64	16	32	32	>64	>64	64	64	48	>64
*S. aureus* ATCC 25923	>64	>64	>64	>64	32	64	>64	>64	>64	>64	32	64
*E. coli* EURL‐VTEC A07 EPEC:O111	16	8	2	1	2	2	–	–	–	–	–	–
*E. coli* EURL‐VTEC C07 STEC:O157	>64	>64	8	8	2	2	–	–	–	–	–	–
*E. coli* SSI‐NN14 ETEC	2	2	1	1	2	1	–	–	–	–	–	–
*E. coli* EA22 ETEC	2	2	1	1	2	1	–	–	–	–	–	–
*E. coli* SSI‐OO15 EIEC	16	4	1	1	1	1	–	–	–	–	–	–
*E. coli* C679‐12 EAEC:0104	16	8	2	2	2	1	–	–	–	–	–	–

Overall, the highly purified derivatives 278, 281 and 291 displayed 2 fold lower MIC against E. coli BW25113 (Table 3) with respect to the values reported in Table 2 and referred to the same molecules obtained by SPOT synthesis. This difference is most likely due to the lower degree of purity achievable using the latter technique.

The five selected Bac5(1–17) derivatives were further tested against reference and pathogenic strains of *E. coli* as well as ATCC reference strains of clinically relevant Gram‐negative pathogens (Table 3) and the Gram‐positive *S. aureus*. Remarkably, all peptides with substitutions possessed increased antibacterial activity against one or more strains among the pathogenic bacteria tested. They were highly effective against four different virotypes of *E. coli* isolates from patients (Table 3), in most cases showing MIC values (1–2 μM) comparable or lower than that of the reference strain BW25113. Remarkably, the Bac5 272, 278 and 291 derivatives acquired antimicrobial activity against *K. pneumoniae* and *A. baumannii* strains with MIC values ranging from 4 to 24 μM, while strikingly 281 was highly active against *A. baumannii* (MIC 4 μM). *P. aeruginosa* and *S. aureus* were intrinsically resistant to the native Bac5(1–17). However, *P. aeruginosa* was moderately sensitive (MIC 16 μM) to the 278 peptide and weakly sensitive (MIC 32–64 μM) to 272, 281 and 291 peptides. On the other hand, *S. aureus* showed weak sensitivity to 281 and 291 (MIC 32–64 μM) (Table 3). By contrast, the 258 peptide displayed no improvement in antimicrobial activity against these strains, suggesting that the broader spectrum of activity displayed by the other peptides is likely due to their newly acquired membrane‐permeabilizing activity. Interestingly, the improvement of antimicrobial activity (reduction of the MIC) often correlated with a reduction in the MBC.

### Cytotoxicity of Bac5(1–17) Derivatives on MEC‐1 Cells

**Figure 4:**
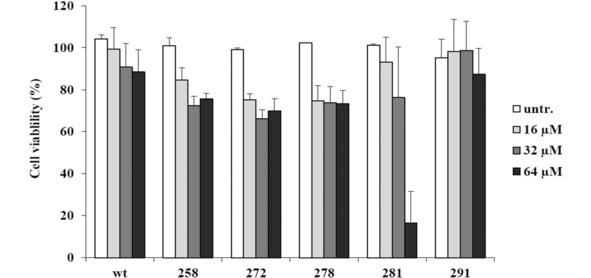
**Toxicity evaluation by MTT assay of selected Bac5(1–17) derivatives toward the human cell line MEC‐1**. The viability of cells treated with peptides is reported in relation to the 100 % viability of a control receiving only sterile water. Most of the peptides at 32 μM display a little decreased cell viability values with respect to those reported in Table 2. Little discrepancies are most likely due to the higher purity of the peptides used in this experiment compared to those used in the assays reported in table 2 (SPOT‐synthesis). Error bars are the standard deviations calculated on the average of 3 independent experiments performed in duplicate (n=6).

Given the increased membrane‐permeabilizing activity and broader spectrum of activity observed by the Bac7(1–17) derivatives, concerns were raised as to the possibility of a corresponding increase in the cytotoxicity of the peptides. To address this, the viability of MEC‐1 cells was assessed in the presence of high concentrations (16–64 μM) of each peptide. At the highest concentration tested (64 μM), only the 281 peptide remarkably reduced cell viability, causing an 80 % reduction in viability with respect to the untreated control after 24 h incubation (Figure 4). The other peptides, including the wild type, did not decrease the viability of the cells by more than 20–30 %, compared to the untreated control. Interestingly, the 291 peptide, which like the 281 peptide, displayed high levels of membrane permeabilizing activity, did not reduce cell viability at 32 μM, and exhibited only mild toxic effects (reduction of 5 % of the cell viability) even at 64 μM.

## Discussion

Here we identified the amino acid residues of the PrAMP Bac5(1–17) that are crucial for the inhibition of the bacterial protein synthesis and viability against *E. coli*. Then, we combined selected residue substitutions obtaining Bac5(1–17) derivatives with increased antibacterial potency and displaying broader spectrum of activities.

**Figure 5:**
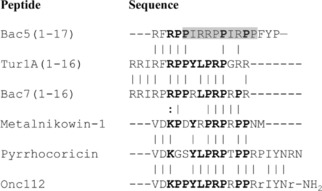
**Sequence alignment between Bac5(1–17) and other ribosome‐targeting PrAMPs**. In bold are the amino acids conserved at least in four different PrAMPs. The grey box indicates the PIRR/PP regions of Bac5(1–17). *D*‐amino acids in Onc112 are indicated by lowercase letters.

Firstly, we showed that Bac5(1–17) does not tolerate single residue substitutions in the two ‐PIRR/PP‐ repeats of the central regions (residues 5–14) which often reduced or abolished the antimicrobial activity of Bac5(1–17) (see Table 1). Substitutions in the −PIRR/PP− sequences impaired in parallel also the inhibition of the protein synthesis, indicating that the central moiety of Bac5(1–17) is crucial for its interaction with the *E. coli* ribosome and therefore for the antimicrobial activity. This region roughly overlaps with the PR‐rich region, shared by many PrAMPs (Figure 5), which targets the exit tunnel of the bacterial ribosome.7b Ala‐scans performed on pyrrhocoricin^15^ and oncocin O1^16^ indicated that residues present in this conserved region are important or crucial for their antimicrobial activity. Mutations I6W and P9R are the only substitutions falling in this Bac5(1–17) region which did not cause loss of activity (Table 1). P9R substitution makes Bac5(1–17) more similar to the other PrAMPs showing a conserved arginine in that position (Figure 5), I6W introduces a sterically bulky group in a position corresponding to a tyrosine or arginine in the other AMPs. Structural studies will be necessary to understand the meaning of these modifications.

We showed previously that the shortening of the Bac5(1–17) sequence affected its antimicrobial activity11b, 14 leading to the assumption that the N‐ and C‐termini were non‐modifiable without a dramatic loss of activity. However, here we found that the ends of Bac5(1–17) sequence can be changed, sometimes even improving the antibacterial activity. Generally, residue substitutions with R or W in the peptide termini fostered the antibacterial potency of Bac5(1–17). Their simultaneous introduction into new peptides sometimes resulted in further improvements of the antimicrobial activity. It is worth noting that these improvements are most likely due to a gain in the capability of the PrAMPs to affect membrane stability. This effect increased progressively from the non‐permeabilizing Bac5(1–17) and B5 peptide 258, to the more permeabilizing and more potent 281 and 291 derivatives (Table 3, Figure 3). The peptides 272 and 278 fell in‐between, acquiring membrane‐destabilizing activity in the range of concentrations tested. In each case, the membrane‐destabilizing effects of the more effective 281 and 291 peptides are far from that exhibited by the peptide antibiotics colistin, used as a reference for a potent lytic antibacterial compound.^17^ However this could be sufficient to promote the efflux of ions and metabolites from the bacterial cells negatively affecting the viability of bacteria.

Interestingly, although these peptides gained a moderate activity on the membrane, they also maintained an unchanged efficacy in targeting the bacterial protein synthesis. Bac5 peptide 281 was the only peptide that exhibited a significantly decreased inhibitory effect on the protein synthesis, maintaining however a relevant antimicrobial potency, that mainly relies on its permeability mechanism. Apart from this exception, the other four peptides might exert a dual mode of action, since they could both cross the bacterial membrane, as suggested by their reduced dependency on the bacterial transporter SbmA, efficiently inhibiting protein synthesis and destabilizing at the same time the bacterial membrane. The latter effect is more relevant at high concentrations. A dual mode of action (membrane destabilization and protein synthesis inhibition) would represent an added value for the antimicrobial potential of these peptides because it makes the selection of bacterial resistance more difficult.

The five selected Bac5(1‐17) derivatives also display a wider spectrum of activity, becoming active against pathogenic strains of *A. baumanni*, *K. pneumoniae* and *E. coli*. The antibacterial activity was tested by using the antibiotic‐reference medium MHB without any dilution and no other “poor” media as often reported in the literature.^18^ This was done to increase the robustness of these results but also to use a standardized method, to make the comparison with other antimicrobials possible. Bac5 derivatives are likely to exploit the SbmA homologue of *E. coli* to enter bacterial cells of *A. baumanni* and *K. pneumoniae* 7b but this is not sufficient to explain their activity, albeit weak, against *P. aeruginosa* and *S. aureus*, both lacking SbmA. A membrane‐destabilizing activity that provides SbmA‐independent mechanism has already been described in *P. aeruginosa* for the mammalian PrAMP Bac7(1‐35) and for the insect‐derived Apideaecins.^19^ This would be in agreement with the membrane‐destabilizing effect observed in this study. Further studies will be necessary to elucidate the mode of action of these peptides on bacterial species other than *E. coli*.

The improvement in antimicrobial potency and activity spectrum of Bac5(1‐17) derivatives is not followed by a parallel increase in toxicity towards eukaryotic cells. Toxicity was only observed at concentrations well above the MIC and MBC values for 4 out of the 5 peptides. The peptide 281 was the only one displaying considerable toxicity and this may be due to its distinct mode of action to permeabilize membranes, and hence to be less selective for bacteria. By contrast, the 291 peptide was not only potent, but also non‐toxic, even at 64 μM, and thus could be promising as a lead compound for development of new drugs.

## Conclusions

In conclusion, we identified Bac5(1–17) derivatives that combine a broad spectrum of activity and a low MIC against relevant pathogens with low cytotoxicity. These peptides are as effective against *E. coli* and other pathogens as the most potent known Bac5 fragment, *i. e*. the Bac5(1–25),11b but have the advantage of being shorter, having a wider activity spectrum as well as a dual mechanism of action. The optimization of mammalian PrAMPs by SPOT‐synthesis provided small, less expensive and more effective molecules to be developed as second‐generation antibiotics.

## Experimental Section

### Peptide Synthesis

Peptides libraries were prepared by SPOT‐synthesis using a MultiPep RSI peptide synthesizer (Intavis, Germany), by adapting reported protocols.^12^ The cellulose membrane (10 cm×15 cm) was functionalised overnight in 0.2 M Fmoc‐Gly‐OH (Aldrich), 0.24 M N,N′‐diisopropylcarbodiimide (DIC, Fluka) and 0.4 M N‐methylimidazole (NMI, Aldrich) in dimethylformamide (DMF, VWR). Then, the glycine was de‐protected in 20 % piperidine (v/v, Acros Organics) in DMF (20 min+10 min). Peptide synthesis was automatically performed at discrete spots using 9‐fluorenyl‐methoxycarbonyl/tert‐butyl (Fmoc/tBu) strategy. For the first cycle, a spotting volumes of 0.8 μl was used, then increased to 0.9 μl for the following cycles. After the synthesis and final deprotection an overnight incubation was performed in a saturated ammonia gas atmosphere to cleave the peptide amides from the solid support. Yield and quality of the synthesis were determined using HPLC/MS analysis on control peptides at different positions on the membranes. Individual SPOTs (Ø=6 mm) were punched‐out from the membrane, transferred into a round‐bottomed sterile 96‐well polypropylene microtiter plate and incubated overnight at room temperature in 200 μl of sterile water. Peptides concentration was measured by absorbance at 280 nm using a NanoDrop 1000 spectrophotometer. Control peptides were subjected to analytical RP‐HPLC on a Shim‐pack VP‐ODS column (120 Å, 150×4.6 mm, Shimadzu) using a LC2010AHT system (Shimadzu) and 0.1 % (v/v) TFA in H_2_O (HPLC‐grade, VWR, solvent A) and 0.1 % (v/v) TFA in acetonitrile (HPLC‐grade, VWR, solvent B). A flow rate of 1 mL min^−1^ was applied to a linear gradient of 5 % to 70 % solvent B in 32.5 min with an initial 3‐min isocratic equilibration. The crude control peptides were <60 % pure. To remove TFA, peptides were re‐suspended in 200 μl of 10 mM HCl and re‐lyophilised. Afterwards the peptide pellets were resuspended in 50 μl of sterile water, quantified at 214 nm by a Nanodrop 2000. The in house‐made program ConCalc was used to calculate the molar coefficient at 214 nm of each peptide using the values reported.^20^ Bac5(1–17), 272, 278, 281 and 291 peptides (≥95 % pure) were purchased from NovoPro (China) and their identity was checked by mass spectrometry. These peptides were lyophilized from 10 mM HCl three times to remove TFA and quantified spectrophotometrically as above.

### Bacterial Culture

The following bacterial strains were used: *Escherichia coli* BW25113, *E. coli* BW25113Δ*sbmA* (JW0368‐1, KEIO collection^21^); *Acinetobacter baumannii* ATCC 19606, *Klebsiella pneumoniae* ATCC 700603, *Pseudomonas aeruginosa* ATCC 27853 and *Staphylococcus aureus* ATCC 25923. The pathogenic *E. coli* EURL‐VTEC A07, *E. coli* EURL‐VTEC C07, *E. coli* SSI‐NN14, *E. coli* EA22, *E. coli* SSI‐OO15 and *E. coli* C679‐12 strains were generously provided by the European Union Reference Laboratory for *E. coli*, Department of Food Safety, Nutrition and Veterinary Public Health, Istituto Superiore di Sanità, Rome, Italy. All the strains were grown overnight in Müller‐Hinton broth (MHB, Difco) at 37 °C, with shaking (140 rpm). The day after, the overnight bacterial cultures were diluted approx. 1 : 40 in fresh MHB and incubated at 37 °C with shaking (140 rpm) until an OD_600nm_ of ≈0.3 was reached. *E. coli* BW25113Δ*sbmA* was grown in the presence of 50 μg mL^−1^ kanamycin.

### Minimum Inhibitory Concentration (MIC) Determination

Peptides were diluted in 100 μL of MHB to the concentration of 128 μM, dispensed in the first wells of a 96‐wells microtiter plate (round‐bottom) and serially two‐fold diluted with MHB into successive wells in a final volume of 50 μl. Subsequently, 50 μL of a bacterial suspension (5×10^5^ CFU mL^−1^) were added to each well, halving the final concentration of bacteria and peptides. Bacteria were added to MHB only as bacterial growth‐control, whereas 100 μl of MHB were used to check the medium sterility. The plate was incubated overnight at 37 °C (≈18 hours) and sealed with parafilm to minimise evaporation. The MIC was calculated as the first clear well without bacterial growth. If barely detectable growth was present, the OD_600nm_ of the plate was measured by a multiplate reader Tecan (software Sunrise). The MIC was then calculated as the well whose OD_620_ was≤1 % of that of the untreated growth control.

### Cytotoxicity Assay

The tetrazolium salts test (MTT) was used. Cells of the human line of B lymphocytes precursors MEC‐1 were exposed to peptides and, as a control, to sterile water. RPMI (Sigma)+10 % Fetal Bovine Serum (Sigma)+2 mM Glutamine+100 U mL^−1^ penicillin+100 μg mL^−1^ streptomycin was used as growth medium. Serial two‐fold dilutions of the peptides were prepared in a 96‐ flat‐bottom microtiter plate (Euroclone) in a final volume of 50 μL of cell growth medium. Cells were counted using a Bürker chamber, then 50 μL of medium containing 10^5^ cells were added to each well, to reach a final volume of 100 μL. Sterile water instead of peptides was used as a control. Cells were incubated for 20 hours at 37 °C and 5 % CO_2_. 25 μL of a sterile MTT solution (Sigma) were the added to each well to the final concentration of 1 mg mL^−1^ in PBS. Following 4 hours of incubation in the dark at 37 °C under 5 % CO_2_, 100 μL of 10 % w/v Igepal (Sigma‐ Aldrich) in 10 mM HCl were added to each well. The plate was incubated overnight at 37 °C then the OD_570nm_ of the wells was measured using a plate‐reader spectrophotometer Nanoquant infinite M200pro (Tecan). To calculate cytotoxicity, the value of OD_570nm_ of the treated samples was compared with that of the untreated control.

### Assessment of Bacterial Membrane Integrity

The bacterial cell integrity was controlled evaluating the propidium iodide (PI) uptake by flow‐cytometry on a Cytomics FC 500 (Beckman‐Coulter). 10^4^ bacterial cells were acquired for each measurement. Mid‐log phase *E. coli* BW25113 cultures diluted to 10^6^ CFU mL^−1^ in MHB were exposed for 30 min to 4 μM and 16 μM peptides at 37 °C in the presence of 10 μg mL^−1^ PI. Membrane permeabilization was calculated as the % PI‐positive cells, and membrane damage measuring the mean fluorescence intensity (MFI). An untreated control received sterile water instead of peptides. By contrast, bacteria were treated with 1 μM colistin as a permeabilizing control. Data are reported as the average ± standard deviation.

### *In vitro* Transcription/Translation Assay

*E. coli* lysate‐based transcription‐translation coupled assays (RTS100, Biotech Rabbit) were performed as described previously.7c, 8a, 8b 6 μL reactions, with or without peptide were mixed according to the manufacturer's instructions and incubated for 1 h at 30 °C with shaking (750 rpm). The reaction was then stopped by adding 5 μL kanamycin (50 μg mL^−1^). All samples were diluted with 40 μL of Luciferase assays substrate (Promega) into a white 96‐well chimney flat bottom microtiter plate (Greiner). The luminescence was then measured using a Tecan Infinite M200 plate reader. The relative values were determined by defining the luminescence value of the sample without inhibitor as 100 %. All the experiments were performed as independent triplicates.

## Conflict of interest

K. Hilpert and D. P. W. Collis are part of the company Tika Diagnostic, London, UK.

## Supporting information

As a service to our authors and readers, this journal provides supporting information supplied by the authors. Such materials are peer reviewed and may be re‐organized for online delivery, but are not copy‐edited or typeset. Technical support issues arising from supporting information (other than missing files) should be addressed to the authors.

Supplementary
