# Supplementary material for: Proline‐Rich Peptides with Improved Antimicrobial Activity against E. coli, K. pneumoniae, and A. baumannii
Source: ChemMedChem. 2019 Nov 14;14(24):2025–33. doi: 10.1002/cmdc.201900465 (PMC6973051; doi:10.1002/cmdc.201900465)
Supplement: Supplementary file 1 — Supplementary [file CMDC-14-2025-s001.pdf]

CHEMMEDCHEM

Supporting Information

© Copyright Wiley-VCH Verlag GmbH & Co. KGaA, 69451 Weinheim, 2019

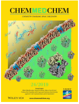

Proline-Rich Peptides with Improved Antimicrobial Activity against *E. coli*, *K. pneumoniae*, and *A. baumannii*

Mario Mardirossian, Riccardo Sola, Bertrand Beckert, Dominic W. P. Collis, Adriana Di Stasi, Federica Armas, Kai Hilpert, Daniel N. Wilson,* and Marco Scocchi*© 2019 The Authors. Published by Wiley-VCH Verlag GmbH & Co. KGaA. This is an open access article under the terms of the Creative Commons Attribution License, which permits use, distribution and reproduction in any medium, provided the original work is properly cited.

BAC7 (1-16)

NovoPro

For enjoyable discovery

CERTIFICATE OF ANALYSIS

**Product Name**	GT31707-1
**Lot No.**	GT31707-1-1207
**Sequence**	RRIRPRPPRLPRPRPR
**Dissolution condition**	100%H2O
**Length**	16AA
**Modification**	N/A
**Molecular Weight (MW)**	2076.49
**Storage**	-20°C

**Test Items**	**Specifications**	**Results**
**MW by MS**	2079.75	Conforms
**Purity by HPLC**	>95%	95.093%
**Peptide Content**	N/A	N/A
**Moisture content**	N/A	N/A
**Acetic acid content**	N/A	N/A
**Appearance**	White to off-white lyophilized powder	Conforms
**Quantity**	10mg	10.0mg

NovoPro Bioscience Inc. (hereafter NovoPro) warrants material of said quality at the time of sale. It is the sole responsibility of the customers to determine the adequacy of all materials for any intended or specific purpose or use. NovoPro's sole obligation is to replace the material up to the extent of the purchase price. This warranty applies only to products in original packaging and does not apply to a product which has been tampered with or altered in any way in or which has been misused or damaged by accident or negligence. All claims must be received writing (by fax or email) within 30 days from date when product arrive at the destination city and failure to do so shall constitute a waiver by customers for any and all such claims.

Certified by: [signature]

Quality Assurance Department

Dec/17/2018

NovoPro Biosciences Inc. VERIFIED Quality Control

BAC7 (1-16)

NovoPro

For enjoyable discovery

Sample Information

Order ID	:GT31707-1
Name	:7wt
Sequence	:RRIRPRPPRLPRPRPR
Lot. No	:GT31707-1-1207
Pump A	:0.1%Trifluoroacetic in 100% water
Pump B	:0.1%Trifluoroacetic in 100% acetonitrile
Total Flow	:1ml/min
Wavelength	:214nm
Analytial column type	:SHIMADZU Inertsil ODS-SP(4.6*250mm*5um)
Dissolution method	:100%H2O
Inj. Volume	: 15ul

Time	Module	Action	Value
0.01	Pumps	B.Conc	2
30.00	Pumps	B.Conc	65
33.00	Pumps	B.Conc	100
38.00	Pumps	B.Conc	100
40.00	Pumps	B.Conc	2
50.00	Controller	Stop	

Chromatogram

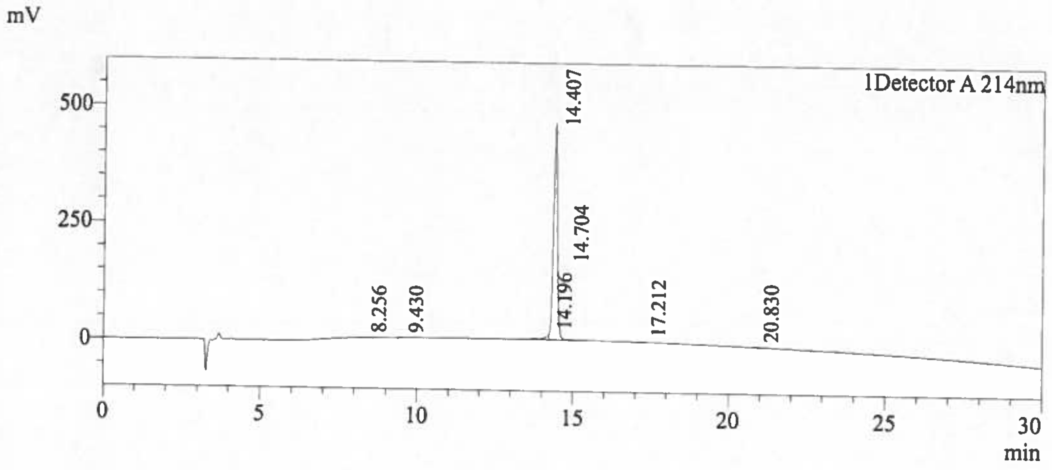

Peak Table

Detector A 214nm

Peak#	Ret. Time	Area	Height	Area%
1	8.256	3446	459	0.083
2	9.430	4244	599	0.102
3	14.196	136202	8350	3.268
4	14.407	3963790	463442	95.093
5	14.704	41038	2488	0.985
6	17.212	6074	934	0.146
7	20.830	13528	1143	0.325
Total		4168322	477414	100.000

NovoPro

MS Spectrum

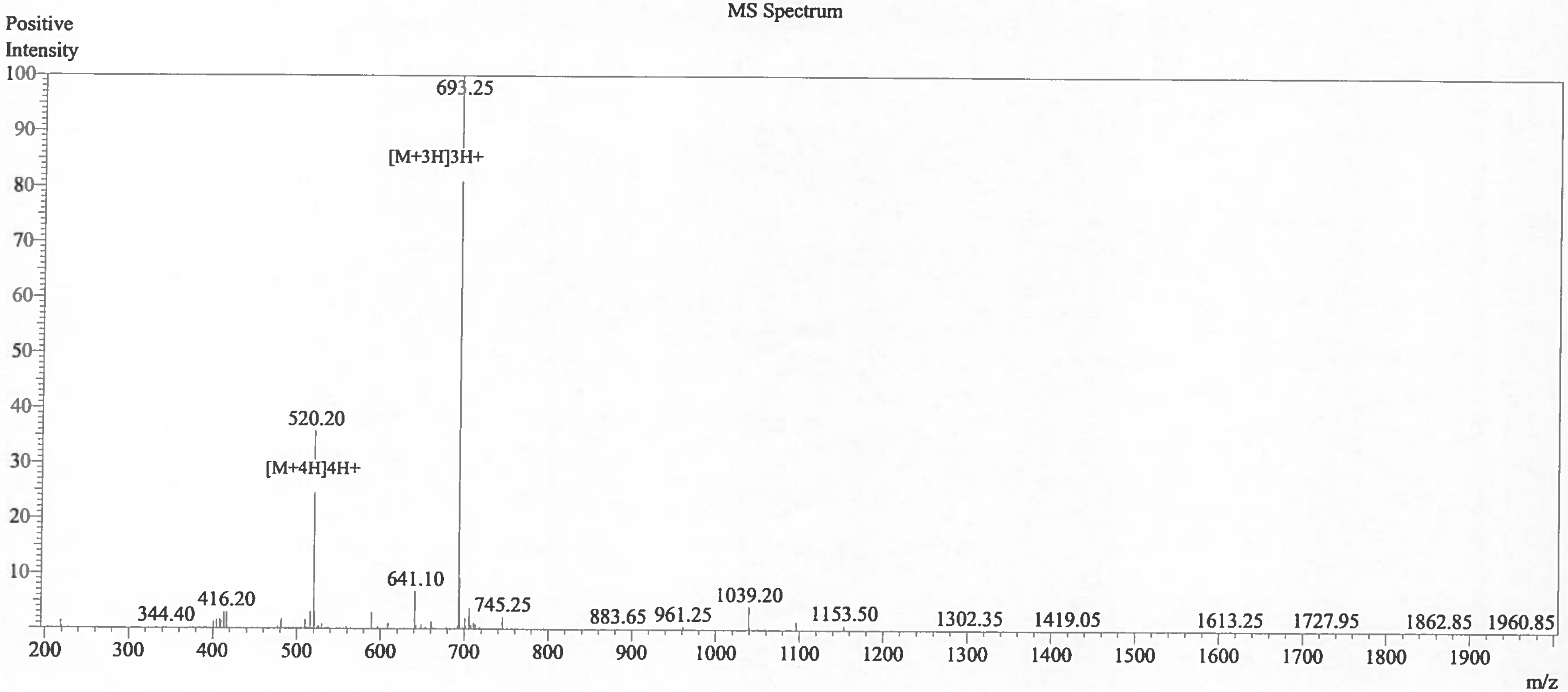

Sample Information

Dissolution method	:5%HAC+8%ACN+87%H2O	Interface	:ESI	Prerod Bias	:+4.5kv
Date Acquired	:2018/12/15 08:50:02	Nebulizing Gas Flow	:1.50L/min	Detector	:-0.2kv
Injection Volume	:0.2ul	CDL Temp	:250C	T.Flow	:0.2ml/min
Block Temp	:200	CDL Volt	:0v	B.conc	:50%H2O/50%MEOH

Order ID	:GT31707-1
Name	:7wt
Sequence	:RRIRPRPPRLPRPRPR
Lot.No	:GT31707-1-1207
Theoretical	:2076.49
Observed	:2076.75

B7 (1-16)

NovoPro

For enjoyable discovery

CERTIFICATE OF ANALYSIS

**Product Name**	GT31707-2
**Lot No.**	GT31707-2-1207
**Sequence**	WRIRPRPPRLPRPRPR
**Dissolution condition**	100%H2O
**Length**	16AA
**Modification**	N/A
**Molecular Weight (MW)**	2106.52
**Storage**	-20°C

Test Items	Specifications	Results
**MW by MS**	2106.60	Conforms
**Purity by HPLC**	>95%	95.034%
**Peptide Content**	N/A	N/A
**Moisture content**	N/A	N/A
**Acetic acid content**	N/A	N/A
**Appearance**	White to off-white lyophilized powder	Conforms
**Quantity**	10mg	10.0mg

NovoPro Bioscience Inc. (hereafter NovoPro) warrants material of said quality at the time of sale. It is the sole responsibility of the customers to determine the adequacy of all materials for any intended or specific purpose or use. NovoPro's sole obligation is to replace the material up to the extent of the purchase price. This warranty applies only to products in original packaging and does not apply to a product which has been tampered with or altered in any way in or which has been misused or damaged by accident or negligence. All claims must be received writing (by fax or email) within 30 days from date when product arrive at the destination city and failure to do so shall constitute a waiver by customers for any and all such claims.

Certified by: [signature]

Quality Assurance Department

Dec/18/2018

NovoPro Biosciences Inc. VERIFIED Quality Control

242

NovoPro

For enjoyable discovery

Sample Information

Order ID	:GT31707-2		
Name	:242		
Sequence	:WRIRPRPPRLPRPRPR		
Lot. No	:GT31707-2-1207		
Pump A	:0.1%Trifluoroacetic in 100% water		
Pump B	:0.1%Trifluoroacetic in 100% acetonitrile		
Total Flow	:1ml/min		
Wavelength	:214nm		
Analytial column type	:SHIMADZU Inertsil ODS-SP(4.6*250mm*5um)		
Dissolution method	:100%H2O		
Inj. Volume	: 15ul		
Time	Module	Action	Value
0.01	Pumps	B.Conc	2
30.00	Pumps	B.Conc	65
33.00	Pumps	B.Conc	100
38.00	Pumps	B.Conc	100
40.00	Pumps	B.Conc	2
50.00	Controller	Stop	

Chromatogram

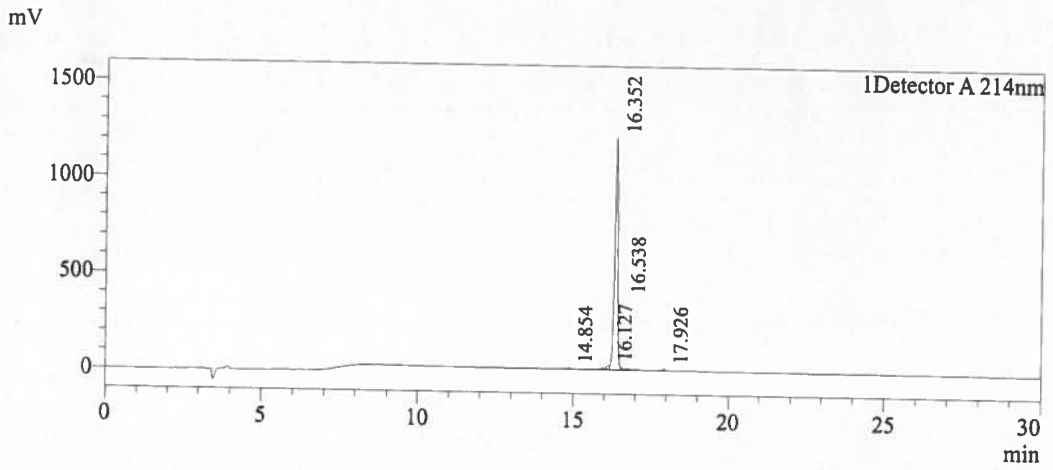

Peak Table

Detector A 214nm

Peak#	Ret. Time	Area	Height	Area%
1	14.854	23671	4689	0.277
2	16.127	200372	19148	2.343
3	16.352	8126260	1203262	95.034
4	16.538	138650	9886	1.621
5	17.926	61947	10015	0.724
Total		8550900	1246999	100.000

NovoPro

242

MS Spectrum

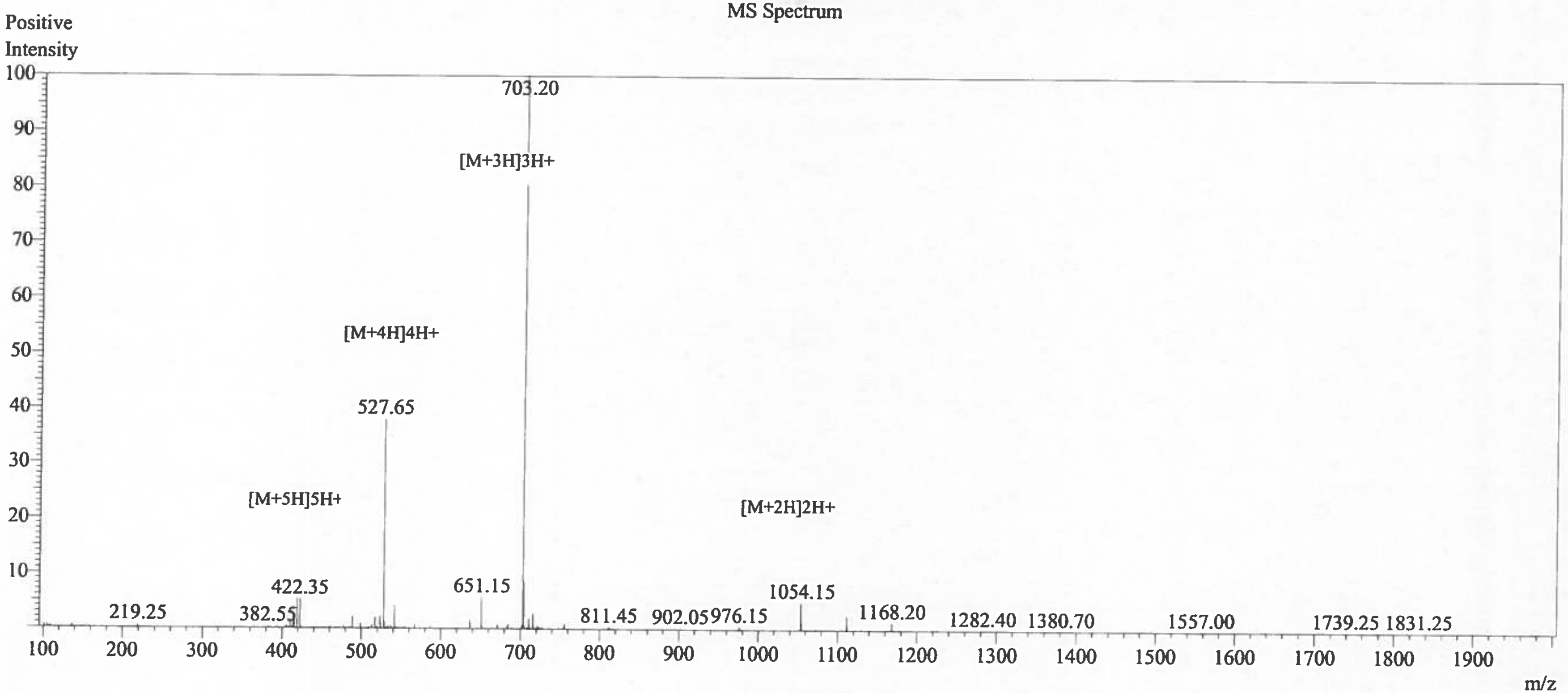

Sample Information

Dissolution method	:5%HAC+8%ACN+87%H2O	Interface	:ESI	Prerod Bias	:+4.5kv
Date Acquired	:2018/12/17 09:24:40	Nebulizing Gas Flow	:1.50L/min	Detector	:-0.2kv
Injection Volume	:0.2ul	CDL Temp	:250C	T.Flow	:0.2ml/min
Block Temp	:200	CDL Volt	:0v	B.conc	:50%H2O/50%MEOH

Order ID	:GT31707-2
Name	:242
Sequence	:WRIRPRPPRLPRPRPR
Lot.No	:GT31707-2-1207
Theoretical	:2106.52
Observed	:2106.60

244

NovoPro

For enjoyable discovery

CERTIFICATE OF ANALYSIS

**Product Name**	GT31707-3
**Lot No.**	GT31707-3-1207
**Sequence**	RRIRPRPPRLPRPRWR
**Dissolution condition**	100%H2O
**Length**	16AA
**Modification**	N/A
**Molecular Weight (MW)**	2165.59
**Storage**	-20°C

**Test Items**	**Specifications**	**Results**
**MW by MS**	2165.70	Conforms
**Purity by HPLC**	>95%	95.024%
**Peptide Content**	N/A	N/A
**Moisture content**	N/A	N/A
**Acetic acid content**	N/A	N/A
**Appearance**	White to off-white lyophilized powder	Conforms
**Quantity**	10mg	10.0mg

NovoPro Bioscience Inc. (hereafter NovoPro) warrants material of said quality at the time of sale. It is the sole responsibility of the customers to determine the adequacy of all materials for any intended or specific purpose or use. NovoPro's sole obligation is to replace the material up to the extent of the purchase price. This warranty applies only to products in original packaging and does not apply to a product which has been tampered with or altered in any way in or which has been misused or damaged by accident or negligence. All claims must be received writing (by fax or email) within 30 days from date when product arrive at the destination city and failure to do so shall constitute a waiver by customers for any and all such claims.

Certified by: [signature]

Quality Assurance Department

Dec/13/2018

NovoPro Biosciences Inc. VERIFIED Quality Control

244

NovoPro

For enjoyable discovery

Sample Information

Order ID	:GT31707-3
Name	:244
Sequence	:RRIRPRPPRLPRPRWR
Lot. No	:GT31707-3-1207
Pump A	:0.1%Trifluoroacetic in 100% water
Pump B	:0.1%Trifluoroacetic in 100% acetonitrile
Total Flow	:1ml/min
Wavelength	:214nm
Analytial column type	:SHIMADZU Inertsil ODS-SP(4.6*250mm*5um)
Dissolution method	:100%H2O
Inj. Volume	: 25ul

Time	Module	Action	Value
0.01	Pumps	B.Conc	2
30.00	Pumps	B.Conc	65
33.00	Pumps	B.Conc	100
38.00	Pumps	B.Conc	100
40.00	Pumps	B.Conc	2
50.00	Controller	Stop	

Chromatogram

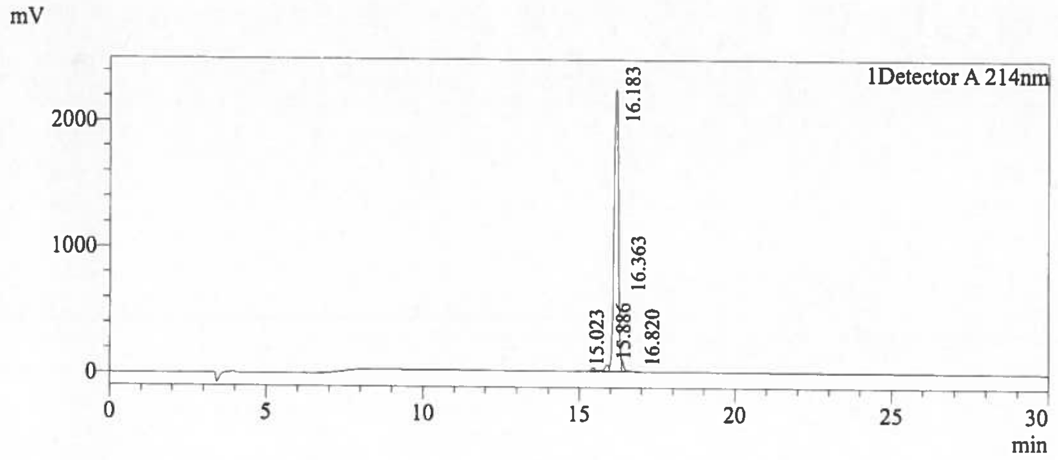

Peak Table

Detector A 214nm

Peak#	Ret. Time	Area	Height	Area%
1	15.023	7971	1151	0.035
2	15.886	698335	51578	3.068
3	16.183	21628832	2235071	95.024
4	16.363	408812	89035	1.796
5	16.820	17521	3263	0.077
Total		22761470	2380099	100.000

NovoPro

244

MS Spectrum

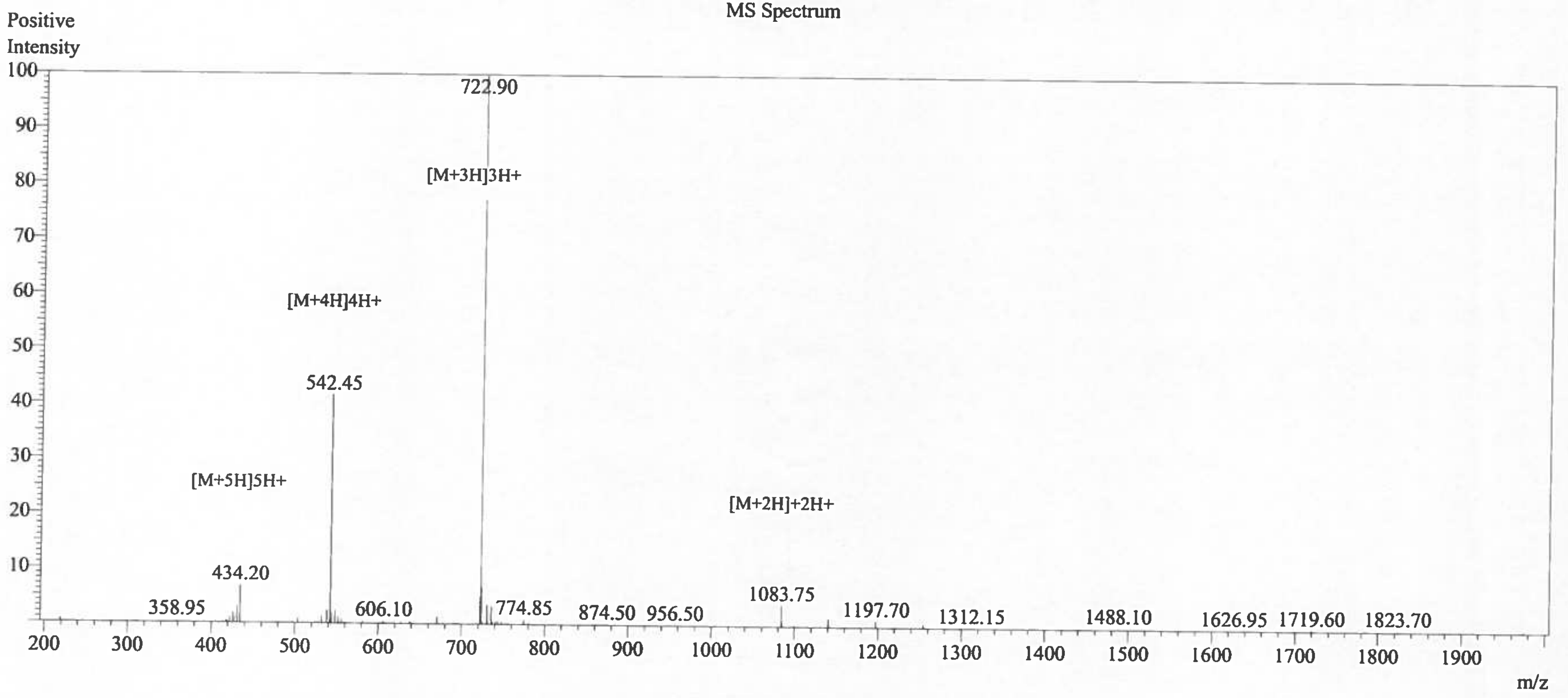

Sample Information

Dissolution method	:5%HAC+8%ACN+87%H2O	Interface	:ESI	Prerod Bias	:+4.5kv
Date Acquired	:2018/12/15 08:54:54	Nebulizing Gas Flow	:1.50L/min	Detector	:-0.2kv
Injection Volume	:0.2ul	CDL Temp	:250C	T.Flow	:0.2ml/min
Block Temp	:200	CDL Volt	:0v	B.conc	:50%H2O/50%MEOH

Order ID	:GT31707-3
Name	:244
Sequence	:RRIRPRPPRLPRPRWR
Lot.No	:GT31707-3-1207
Theoretical	:2165.59
Observed	:2165.70

245

NovoPro

For enjoyable discovery

CERTIFICATE OF ANALYSIS

**Product Name**	GT31707-4
**Lot No.**	GT31707-4-1207
**Sequence**	WRIRPRPPRLPRWRPR
**Dissolution condition**	100%H2O
**Length**	16AA
**Modification**	N/A
**Molecular Weight (MW)**	2195.62
**Storage**	-20°C

**Test Items**	**Specifications**	**Results**
**MW by MS**	2195.55	Conforms
**Purity by HPLC**	>95%	95.754%
**Peptide Content**	N/A	N/A
**Moisture content**	N/A	N/A
**Acetic acid content**	N/A	N/A
**Appearance**	White to off-white lyophilized powder	Conforms
**Quantity**	10mg	10.0mg

NovoPro Bioscience Inc. (hereafter NovoPro) warrants material of said quality at the time of sale. It is the sole responsibility of the customers to determine the adequacy of all materials for any intended or specific purpose or use. NovoPro's sole obligation is to replace the material up to the extent of the purchase price. This warranty applies only to products in original packaging and does not apply to a product which has been tampered with or altered in any way in or which has been misused or damaged by accident or negligence. All claims must be received writing (by fax or email) within 30 days from date when product arrive at the destination city and failure to do so shall constitute a waiver by customers for any and all such claims.

Certified by: [signature]

Quality Assurance Department

Dec/17/2018

NovoPro Biosciences Inc. VERIFIED Quality Control

245

NovoPro

For enjoyable discovery

Sample Information

Order ID	:GT31707-4
Name	:245
Sequence	:WRIRPRPPRLPRWRPR
Lot. No	:GT31707-4-1207
Pump A	:0.1%Trifluoroacetic in 100% water
Pump B	:0.1%Trifluoroacetic in 100% acetonitrile
Total Flow	:1ml/min
Wavelength	:214nm
Analytial column type	:SHIMADZU Inertsil ODS-SP(4.6*250mm*5um)
Dissolution method	:100%H2O
Inj. Volume	: 10ul

Time	Module	Action	Value
0.01	Pumps	B.Conc	2
30.00	Pumps	B.Conc	65
33.00	Pumps	B.Conc	100
38.00	Pumps	B.Conc	100
40.00	Pumps	B.Conc	2
50.00	Controller	Stop	

Chromatogram

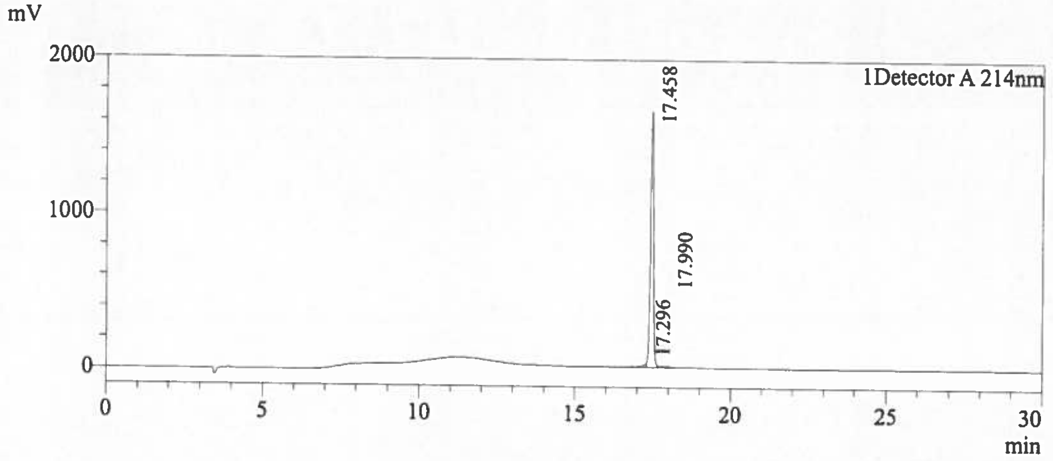

Peak Table

Detector A 214nm

Peak#	Ret. Time	Area	Height	Area%
1	17.296	297604	24647	2.783
2	17.458	10239216	1643273	95.754
3	17.990	156450	8360	1.463
Total		10693271	1676279	100.000

NovoPro

MS Spectrum

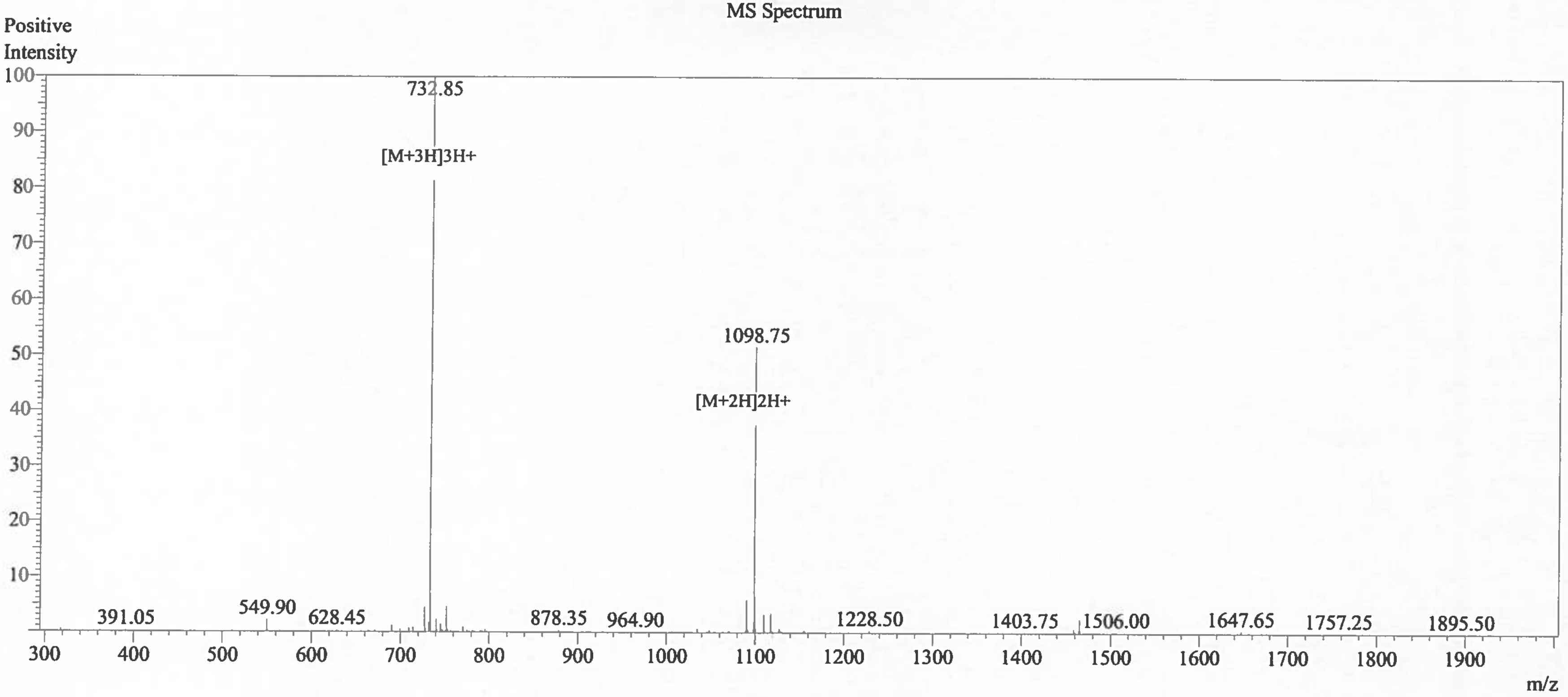

Sample Information

Dissolution method	:5%HAC+8%ACN+87%H2O	Interface	:ESI	Prerod Bias	:+4.5kv
Date Acquired	:2018/12/15 10:12:22	Nebulizing Gas Flow	:1.50L/min	Detector	:-0.2kv
Injection Volume	:0.2ul	CDL Temp	:250C	T.Flow	:0.2ml/min
Block Temp	:200	CDL Volt	:0v	B.conc	:50%H2O/50%MEOH

Order ID	:GT31707-4
Name	:245
Sequence	:WRIRPRPPRLPRWRPR
Lot.No	:GT31707-4-1207
Theoretical	:2195.62
Observed	:2195.55

245

250

NovoPro

For enjoyable discovery

CERTIFICATE OF ANALYSIS

**Product Name**	GT31707-5
**Lot No.**	GT31707-5-1207
**Sequence**	WRIRRRPPRLPRPRWR
**Dissolution condition**	100%H2O
**Length**	16AA
**Modification**	N/A
**Molecular Weight (MW)**	2254.69
**Storage**	-20°C

**Test Items**	**Specifications**	**Results**
**MW by MS**	2255.10	Conforms
**Purity by HPLC**	>95%	95.220%
**Peptide Content**	N/A	N/A
**Moisture content**	N/A	N/A
**Acetic acid content**	N/A	N/A
**Appearance**	White to off-white lyophilized powder	Conforms
**Quantity**	10mg	10.0mg

NovoPro Bioscience Inc. (hereafter NovoPro) warrants material of said quality at the time of sale. It is the sole responsibility of the customers to determine the adequacy of all materials for any intended or specific purpose or use. NovoPro's sole obligation is to replace the material up to the extent of the purchase price. This warranty applies only to products in original packaging and does not apply to a product which has been tampered with or altered in any way in or which has been misused or damaged by accident or negligence. All claims must be received writing (by fax or email) within 30 days from date when product arrive at the destination city and failure to do so shall constitute a waiver by customers for any and all such claims.

Certified by: [signature]

Quality Assurance Department

Dec/13/2018

NovoPro Biosciences Inc. VERIFIED Quality Control

NovoPro

For enjoyable discovery

Sample Information

Order ID	:GT31707-5
Name	:250
Sequence	:WRIRRRPPRLPRPRWR
Lot. No	:GT31707-5-1207
Pump A	:0.1%Trifluoroacetic in 100% water
Pump B	:0.1%Trifluoroacetic in 100% acetonitrile
Total Flow	:1ml/min
Wavelength	:214nm
Analytial column type	:SHIMADZU Inertsil ODS-SP(4.6*250mm*5um)
Dissolution method	:100%H2O
Inj. Volume	: 25ul

Time	Module	Action	Value
0.01	Pumps	B.Conc	2
30.00	Pumps	B.Conc	65
33.00	Pumps	B.Conc	100
38.00	Pumps	B.Conc	100
40.00	Pumps	B.Conc	2
50.00	Controller	Stop	

Chromatogram

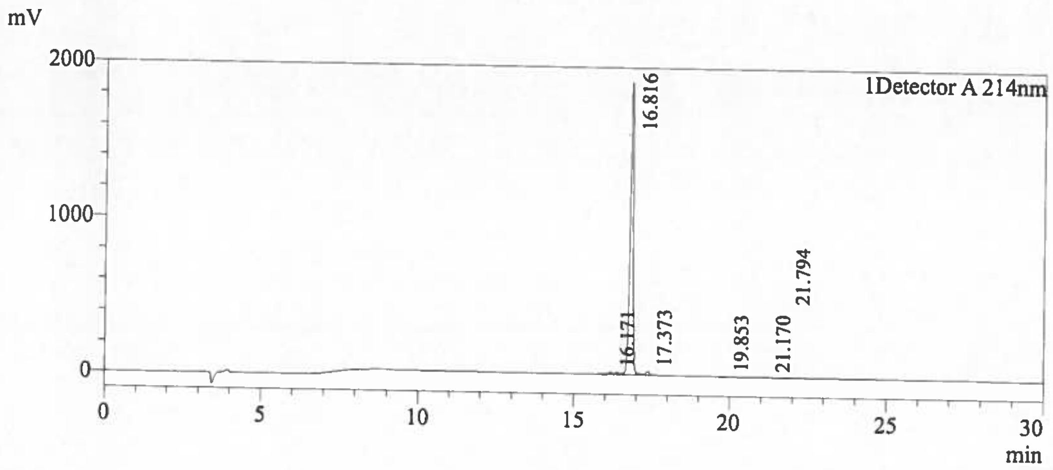

Peak Table

Detector A 214nm

Peak#	Ret. Time	Area	Height	Area%
1	16.171	305472	15438	2.266
2	16.816	12838537	1879442	95.220
3	17.373	315495	25325	2.340
4	19.853	5434	936	0.040
5	21.170	4112	609	0.030
6	21.794	13929	2214	0.103
Total		13482978	1923963	100.000

NovoPro

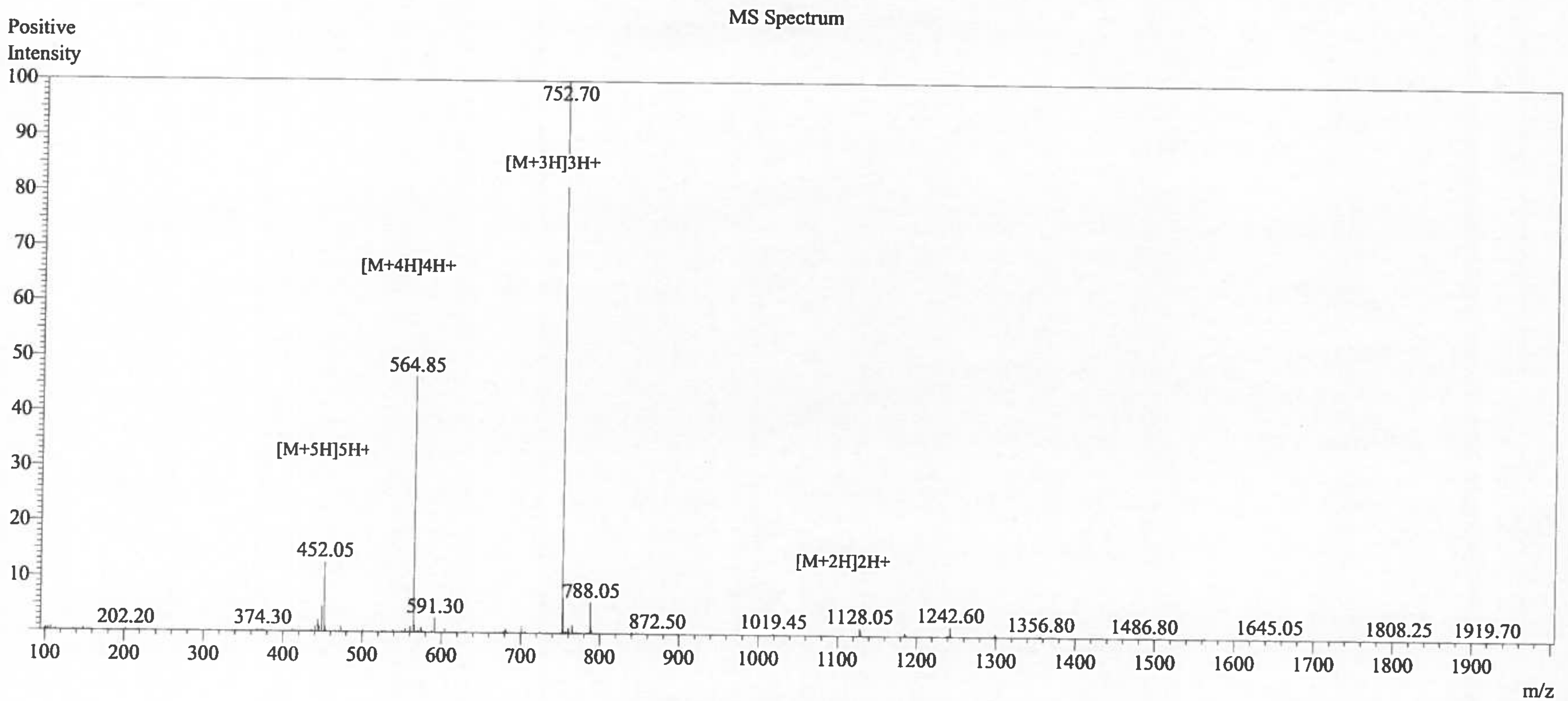

Sample Information

Dissolution method	:5%HAC+8%ACN+87%H2O	Interface	:ESI	Prerod Bias	:+4.5kv
Date Acquired	:2018/12/15 08:58:56	Nebulizing Gas Flow	:1.50L/min	Detector	:-0.2kv
Injection Volume	:0.2ul	CDL Temp	:250C	T.Flow	:0.2ml/min
Block Temp	:200	CDL Volt	:0v	B.conc	:50%H2O/50%MEOH

Order ID	:GT31707-5
Name	:250
Sequence	:WRIRRRPPRLPRPRWR
Lot.No	:GT31707-5-1207
Theoretical	:2254.69
Observed	:2255.10

250

NovoPro

For enjoyable discovery

CERTIFICATE OF ANALYSIS

**Product Name**	GT31707-6
**Lot No.**	GT31707-6-1207
**Sequence**	WRIRRRWPRLPRPRWR
**Dissolution condition**	100%H2O
**Length**	16AA
**Modification**	N/A
**Molecular Weight (MW)**	2343.79
**Storage**	-20°C

**Test Items**	**Specifications**	**Results**
**MW by MS**	2343.50	Conforms
**Purity by HPLC**	>95%	98.386%
**Peptide Content**	N/A	N/A
**Moisture content**	N/A	N/A
**Acetic acid content**	N/A	N/A
**Appearance**	White to off-white lyophilized powder	Conforms
**Quantity**	10mg	10.0mg

NovoPro Bioscience Inc. (hereafter NovoPro) warrants material of said quality at the time of sale. It is the sole responsibility of the customers to determine the adequacy of all materials for any intended or specific purpose or use. NovoPro's sole obligation is to replace the material up to the extent of the purchase price. This warranty applies only to products in original packaging and does not apply to a product which has been tampered with or altered in any way in or which has been misused or damaged by accident or negligence. All claims must be received writing (by fax or email) within 30 days from date when product arrive at the destination city and failure to do so shall constitute a waiver by customers for any and all such claims.

Certified by: [signature]

Quality Assurance Department

Dec/18/2018

NovoPro Biosciences Inc. VERIFIED Quality Control

B7 BEST CAND 254

NovoPro

For enjoyable discovery

Sample Information

Order ID	:GT31707-6
Name	:254
Sequence	:WRIRRRWPRLPRPRWR
Lot. No	:GT31707-6-1207
Pump A	:0.1%Trifluoroacetic in 100% water
Pump B	:0.1%Trifluoroacetic in 100% acetonitrile
Total Flow	:1ml/min
Wavelength	:214nm
Analytial column type	:SHIMADZU Inertsil ODS-SP(4.6*250mm*5um)
Dissolution method	:100%H2O
Inj. Volume	: 10ul

Time	Module	Action	Value
0.01	Pumps	B.Conc	2
30.00	Pumps	B.Conc	65
33.00	Pumps	B.Conc	100
38.00	Pumps	B.Conc	100
40.00	Pumps	B.Conc	2
50.00	Controller	Stop	

Chromatogram

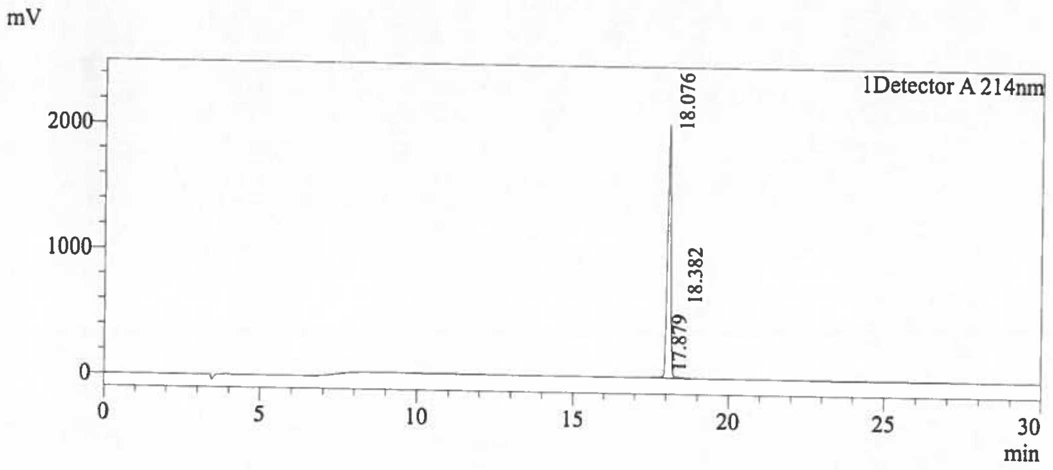

Peak Table

Detector A 214nm

Peak#	Ret. Time	Area	Height	Area%
1	17.879	128223	9487	0.887
2	18.076	14224262	2020394	98.386
3	18.382	105136	9516	0.727
Total		14457621	2039397	100.000

NovoPro

B7 BEST CAND 254

MS Spectrum

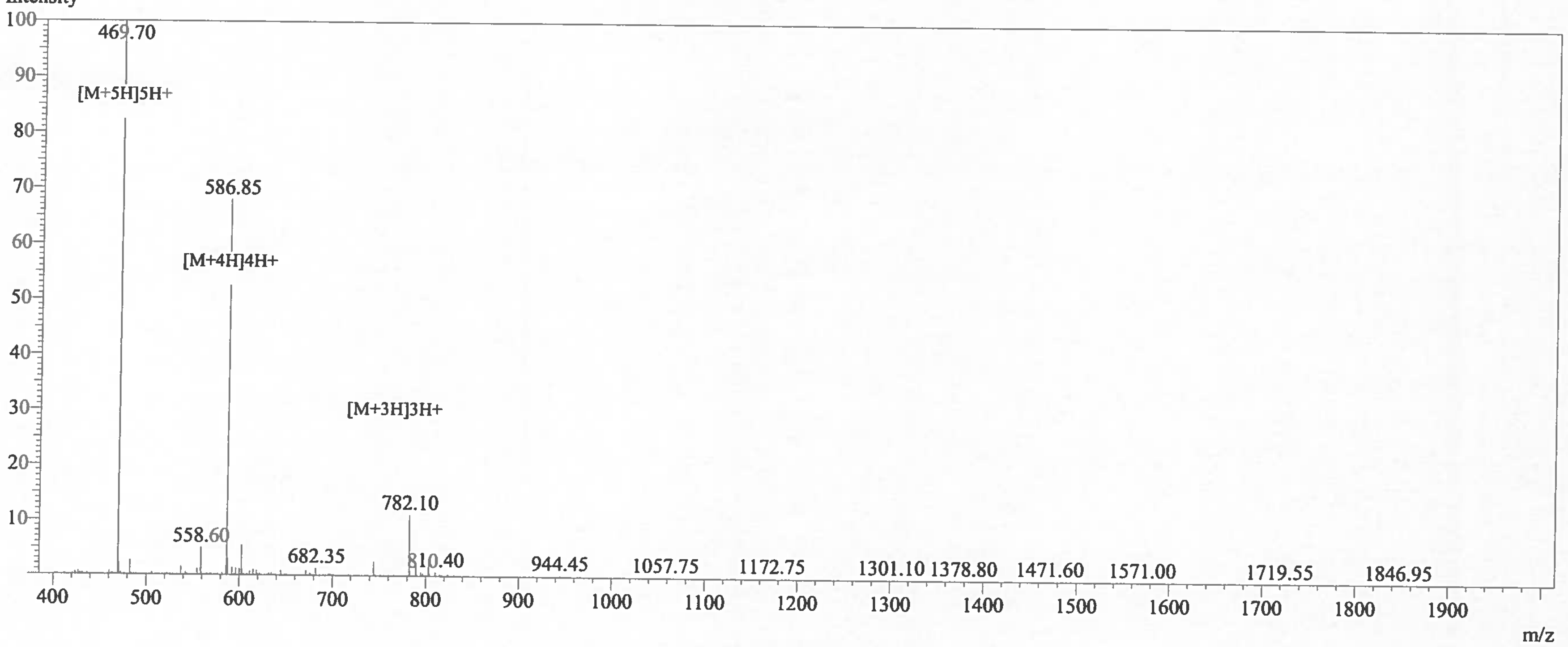

Sample Information

Dissolution method	:5%HAC+8%ACN+87%H2O	Interface	:ESI	Prerod Bias	:+4.5kv
Date Acquired	:2018/12/17 09:05:35	Nebulizing Gas Flow	:1.50L/min	Detector	:-0.2kv
Injection Volume	:0.2ul	CDL Temp	:250C	T.Flow	:0.2ml/min
Block Temp	:200	CDL Volt	:0v	B.conc	:50%H2O/50%MEOH

Order ID	:GT31707-6
Name	:254
Sequence	:WRIRRRWPRLPRPRWR
Lot.No	:GT31707-6-1207
Theoretical	:2343.79
Observed	:2343.50

NovoPro

For enjoyable discovery

CERTIFICATE OF ANALYSIS

BACS (1-17)

**Product Name**	GT31707-7
**Lot No.**	GT31707-7-1207
**Sequence**	RFRPPIRRPPIRPPFYP
**Dissolution condition**	100%H2O
**Length**	17AA
**Modification**	N/A
**Molecular Weight (MW)**	2162.58
**Storage**	-20°C

**Test Items**	**Specifications**	**Results**
**MW by MS**	2162.25	Conforms
**Purity by HPLC**	>95%	95.980%
**Peptide Content**	N/A	N/A
**Moisture content**	N/A	N/A
**Acetic acid content**	N/A	N/A
**Appearance**	White to off-white lyophilized powder	Conforms
**Quantity**	10mg	10.0mg

NovoPro Bioscience Inc. (hereafter NovoPro) warrants material of said quality at the time of sale. It is the sole responsibility of the customers to determine the adequacy of all materials for any intended or specific purpose or use. NovoPro's sole obligation is to replace the material up to the extent of the purchase price. This warranty applies only to products in original packaging and does not apply to a product which has been tampered with or altered in any way in or which has been misused or damaged by accident or negligence. All claims must be received writing (by fax or email) within 30 days from date when product arrive at the destination city and failure to do so shall constitute a waiver by customers for any and all such claims.

Certified by: [signature]

Quality Assurance Department

Dec/18/2018

NovoPro Biosciences Inc. VERIFIED Quality Control

BK5 (1-17)

NovoPro

For enjoyable discovery

Sample Information

Order ID	:GT31707-7
Name	:5wt
Sequence	:RFRPPIRRPPIRPPFYP
Lot. No	:GT31707-7-1207
Pump A	:0.1%Trifluoroacetic in 100% water
Pump B	:0.1%Trifluoroacetic in 100% acetonitrile
Total Flow	:1ml/min
Wavelength	:214nm
Analytial column type	:SHIMADZU Inertsil ODS-SP(4.6*250mm*5um)
Dissolution method	:100%H2O
Inj. Volume	: 20ul

Time	Module	Action	Value
0.01	Pumps	B.Conc	2
30.00	Pumps	B.Conc	65
33.00	Pumps	B.Conc	100
38.00	Pumps	B.Conc	100
40.00	Pumps	B.Conc	2
50.00	Controller	Stop	

Chromatogram

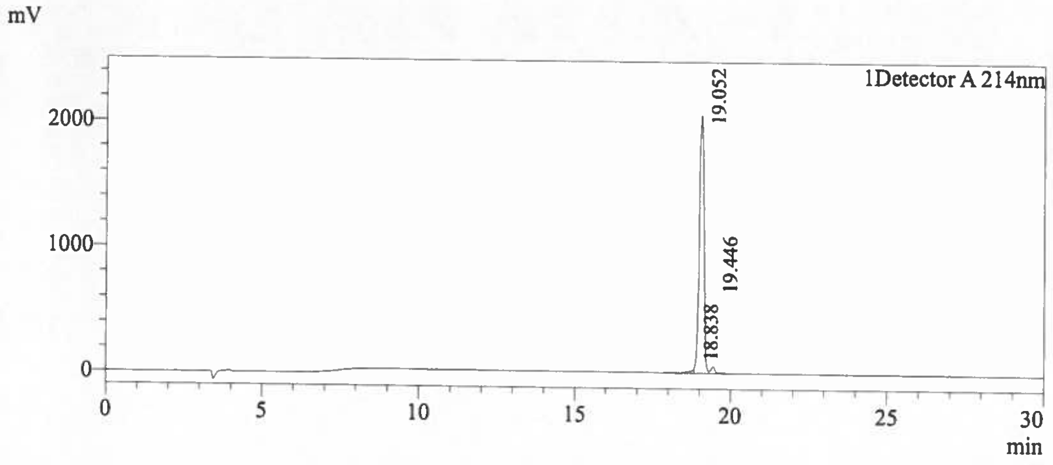

Peak Table

Detector A 214nm

Peak#	Ret. Time	Area	Height	Area%
1	18.838	355307	34250	1.611
2	19.052	21168444	2047613	95.980
3	19.446	531209	53583	2.409
Total		22054959	2135446	100.000

NovoPro

MS Spectrum

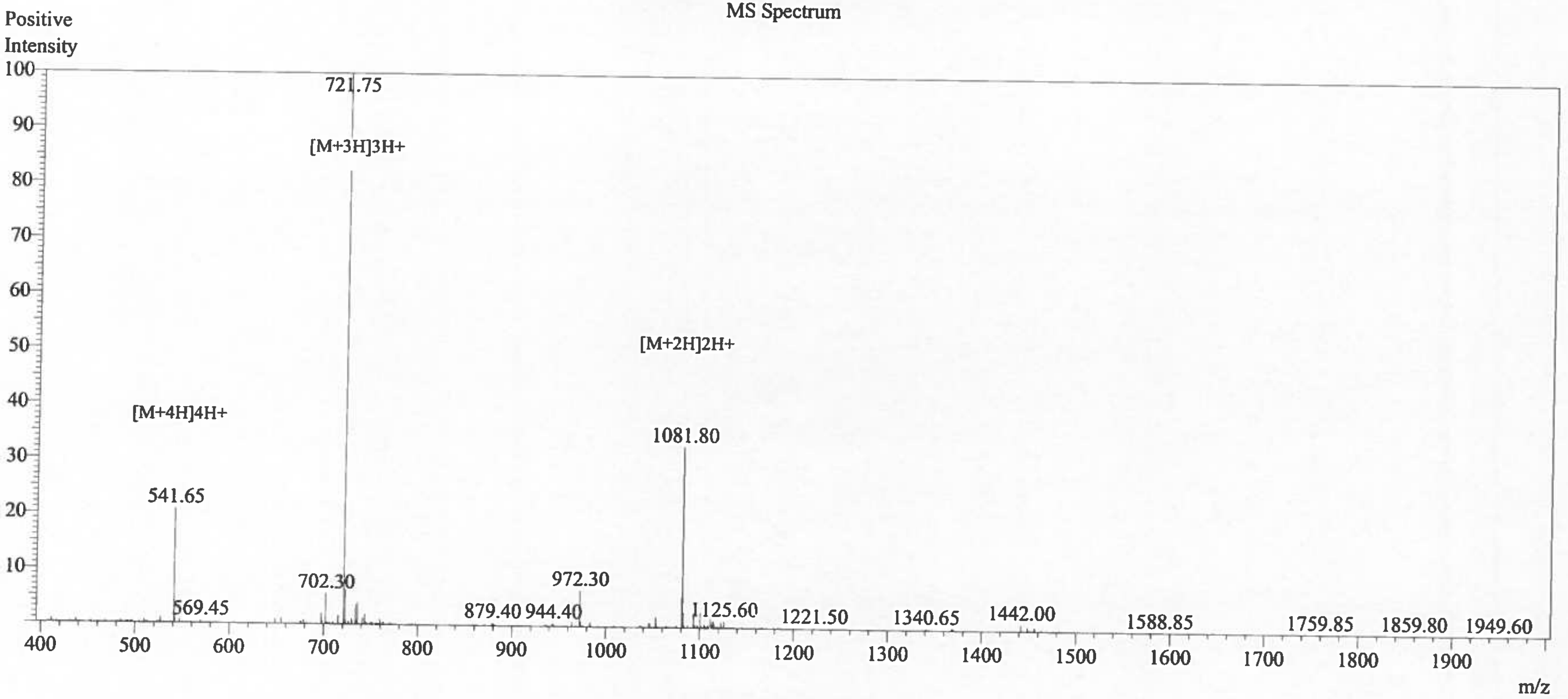

Sample Information

Dissolution method	:5%HAC+8%ACN+87%H2O	Interface	:ESI	Prerod Bias	:+4.5kv
Date Acquired	:2018/12/17 09:12:13	Nebulizing Gas Flow	:1.50L/min	Detector	:-0.2kv
Injection Volume	:0.2ul	CDL Temp	:250C	T.Flow	:0.2ml/min
Block Temp	:200	CDL Volt	:0v	B.conc	:50%H2O/50%MEOH

Order ID	:GT31707-7
Name	:5wt
Sequence	:RFRPPIRRPPIRPPFYP
Lot.No	:GT31707-7-1207
Theoretical	:2162.58
Observed	:2162.25

Bac5(1-17)

258

NovoPro

For enjoyable discovery

CERTIFICATE OF ANALYSIS

**Product Name**	GT31707-8
**Lot No.**	GT31707-8-1207
**Sequence**	RFRPPIRRPPIRPPFYR
**Dissolution condition**	100%H2O
**Length**	17AA
**Modification**	N/A
**Molecular Weight (MW)**	2221.65
**Storage**	-20°C

**Test Items**	**Specifications**	**Results**
**MW by MS**	2221.50	Conforms
**Purity by HPLC**	>95%	97.577%
**Peptide Content**	N/A	N/A
**Moisture content**	N/A	N/A
**Acetic acid content**	N/A	N/A
**Appearance**	White to off-white lyophilized powder	Conforms
**Quantity**	10mg	10.0mg

NovoPro Bioscience Inc. (hereafter NovoPro) warrants material of said quality at the time of sale. It is the sole responsibility of the customers to determine the adequacy of all materials for any intended or specific purpose or use. NovoPro's sole obligation is to replace the material up to the extent of the purchase price. This warranty applies only to products in original packaging and does not apply to a product which has been tampered with or altered in any way in or which has been misused or damaged by accident or negligence. All claims must be received writing (by fax or email) within 30 days from date when product arrive at the destination city and failure to do so shall constitute a waiver by customers for any and all such claims.

Certified by: [illegible]

Quality Assurance Department

Dec/15/2018

NovoPro Biosciences Inc.
VERIFIED
Quality Control

NovoPro

For enjoyable discovery

Sample Information

Order ID	:GT31707-8
Name	:258
Sequence	:RFRPPIRRPPIRPPFYR
Lot. No	:GT31707-8-1207
Pump A	:0.1%Trifluoroacetic in 100% water
Pump B	:0.1%Trifluoroacetic in 100% acetonitrile
Total Flow	:1ml/min
Wavelength	:214nm
Analytial column type	:SHIMADZU Inertsil ODS-SP(4.6*250mm*5um)
Dissolution method	:100%H2O
Inj. Volume	: 25ul

Time	Module	Action	Value
0.01	Pumps	B.Conc	2
30.00	Pumps	B.Conc	65
33.00	Pumps	B.Conc	100
38.00	Pumps	B.Conc	100
40.00	Pumps	B.Conc	2
50.00	Controller	Stop	

Chromatogram

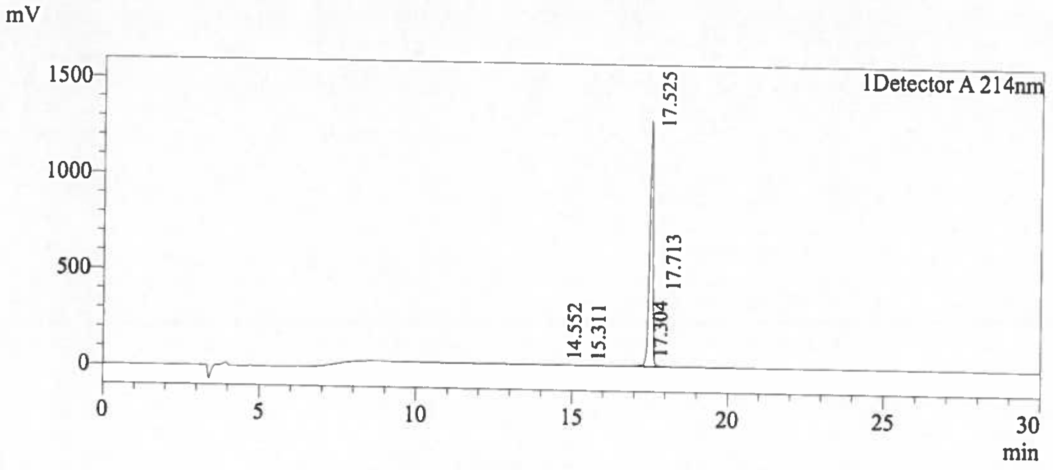

Peak Table

Detector A 214nm

Peak#	Ret. Time	Area	Height	Area%
1	14.552	17087	1152	0.181
2	15.311	5967	71	0.063
3	17.304	100451	11348	1.065
4	17.525	9203914	1279767	97.577
5	17.713	105070	8222	1.114
Total		9432489	1300560	100.000

NovoPro

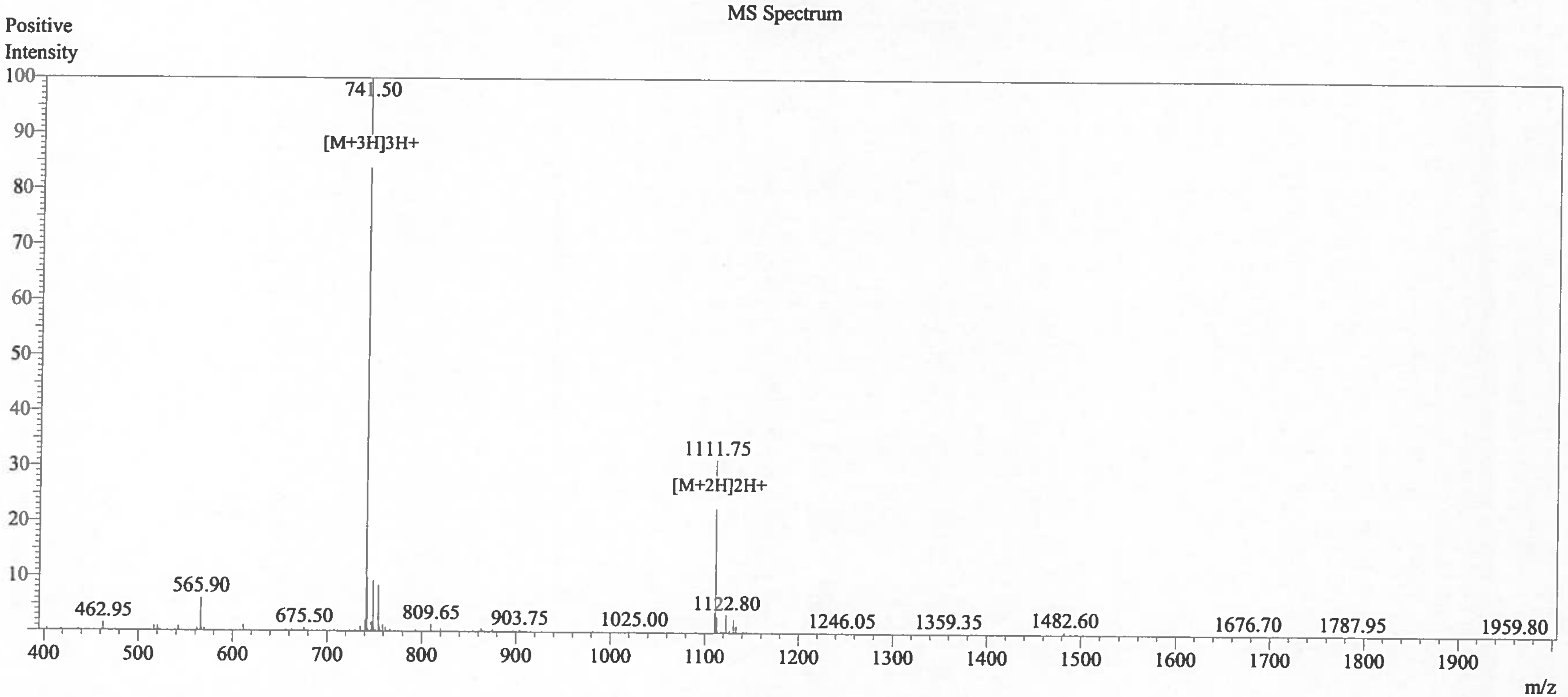

Sample Information

Dissolution method	:5%HAC+8%ACN+87%H2O	Interface	:ESI	Prerod Bias	:+4.5kv
Date Acquired	:2018/12/10 09:20:00	Nebulizing Gas Flow	:1.50L/min	Detector	:-0.2kv
Injection Volume	:0.2ul	CDL Temp	:250C	T.Flow	:0.2ml/min
Block Temp	:200	CDL Volt	:0v	B.conc	:50%H2O/50%MEOH

Order ID	:GT31707-8
Name	:258
Sequence	:RFRPPIRRPPIRPPFYR
Lot.No	:GT31707-8-1207
Theoretical	:2221.65
Observed	:2221.50

258

NovoPro

For enjoyable discovery

CERTIFICATE OF ANALYSIS

**Product Name**	GT31707-9
**Lot No.**	GT31707-9-1207
**Sequence**	RFRWPIRRPPIRPPFYR
**Dissolution condition**	100%H2O
**Length**	17AA
**Modification**	N/A
**Molecular Weight (MW)**	2310.75
**Storage**	-20°C

**Test Items**	**Specifications**	**Results**
**MW by MS**	2310.75	Conforms
**Purity by HPLC**	>95%	95.352%
**Peptide Content**	N/A	N/A
**Moisture content**	N/A	N/A
**Acetic acid content**	N/A	N/A
**Appearance**	White to off-white lyophilized powder	Conforms
**Quantity**	10mg	10.0mg

NovoPro Bioscience Inc. (hereafter NovoPro) warrants material of said quality at the time of sale. It is the sole responsibility of the customers to determine the adequacy of all materials for any intended or specific purpose or use. NovoPro's sole obligation is to replace the material up to the extent of the purchase price. This warranty applies only to products in original packaging and does not apply to a product which has been tampered with or altered in any way in or which has been misused or damaged by accident or negligence. All claims must be received writing (by fax or email) within 30 days from date when product arrive at the destination city and failure to do so shall constitute a waiver by customers for any and all such claims.

Certified by: [signature]

Quality Assurance Department

Dec/15/2018

NovoPro Biosciences Inc. VERIFIED Quality Control

272

NovoPro

For enjoyable discovery

Sample Information

Order ID	:GT31707-9
Name	:272
Sequence	:RFRWPIRRPPIRPPFYR
Lot. No	:GT31707-9-1207
Pump A	:0.1%Trifluoroacetic in 100% water
Pump B	:0.1%Trifluoroacetic in 100% acetonitrile
Total Flow	:1ml/min
Wavelength	:214nm
Analytial column type	:SHIMADZU Inertsil ODS-SP(4.6*250mm*5um)
Dissolution method	:100%H2O
Inj. Volume	: 25ul

Time	Module	Action	Value
0.01	Pumps	B.Conc	2
30.00	Pumps	B.Conc	65
33.00	Pumps	B.Conc	100
38.00	Pumps	B.Conc	100
40.00	Pumps	B.Conc	2
50.00	Controller	Stop	

Chromatogram

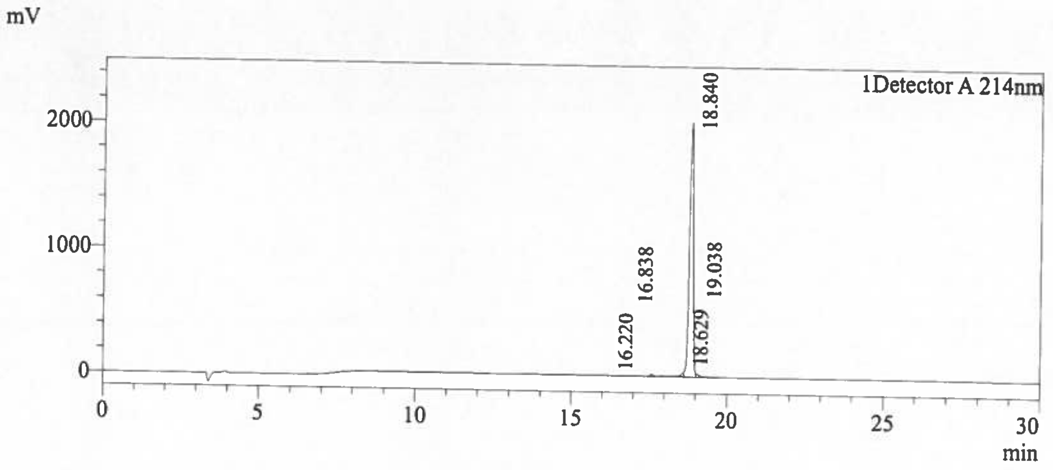

Peak Table

Detector A 214nm

Peak#	Ret. Time	Area	Height	Area%
1	16.220	5908	888	0.035
2	16.838	29460	2056	0.173
3	18.629	512925	33951	3.009
4	18.840	16251510	2033244	95.352
5	19.038	243944	27454	1.431
Total		17043747	2097592	100.000

272

NovoPro

MS Spectrum

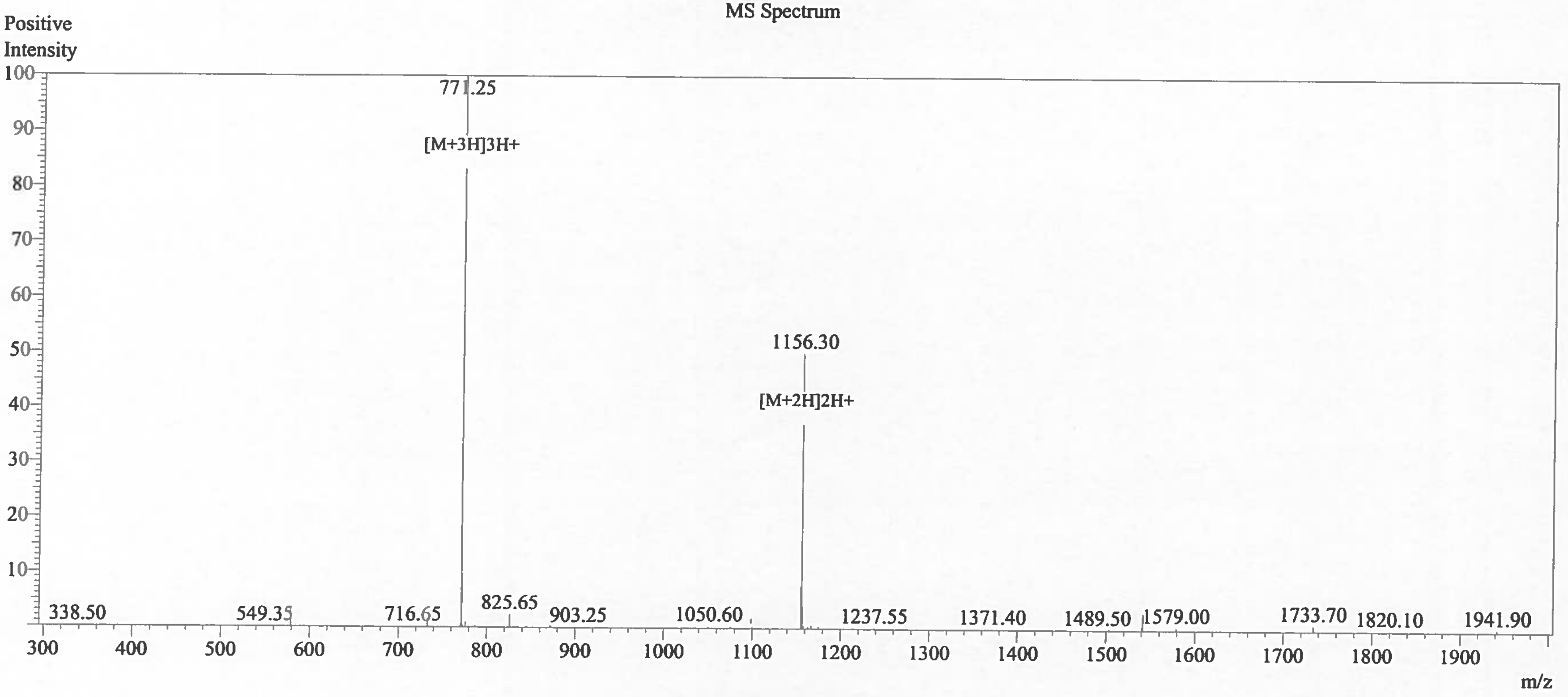

Sample Information

Dissolution method	:5%HAC+8%ACN+87%H2O	Interface	:ESI	Prerod Bias	:+4.5kv
Date Acquired	:2018/12/10 09:13:23	Nebulizing Gas Flow	:1.50L/min	Detector	:-0.2kv
Injection Volume	:0.2ul	CDL Temp	:250C	T.Flow	:0.2ml/min
Block Temp	:200	CDL Volt	:0v	B.conc	:50%H2O/50%MEOH

Order ID	:GT31707-9
Name	:272
Sequence	:RFRWPIRRPPIRPPFYR
Lot.No	:GT31707-9-1207
Theoretical	:2310.75
Observed	:2310.75

278

NovoPro

For enjoyable discovery

CERTIFICATE OF ANALYSIS

**Product Name**	GT31707-10
**Lot No.**	GT31707-10-1207
**Sequence**	RWRWPIRRPPIRPPFYR
**Dissolution condition**	100%H2O
**Length**	17AA
**Modification**	N/A
**Molecular Weight (MW)**	2349.79
**Storage**	-20°C

**Test Items**	**Specifications**	**Results**
**MW by MS**	2349.90	Conforms
**Purity by HPLC**	>95%	95.227%
**Peptide Content**	N/A	N/A
**Moisture content**	N/A	N/A
**Acetic acid content**	N/A	N/A
**Appearance**	White to off-white lyophilized powder	Conforms
**Quantity**	10mg	10.0mg

NovoPro Bioscience Inc. (hereafter NovoPro) warrants material of said quality at the time of sale. It is the sole responsibility of the customers to determine the adequacy of all materials for any intended or specific purpose or use. NovoPro's sole obligation is to replace the material up to the extent of the purchase price. This warranty applies only to products in original packaging and does not apply to a product which has been tampered with or altered in any way in or which has been misused or damaged by accident or negligence. All claims must be received writing (by fax or email) within 30 days from date when product arrive at the destination city and failure to do so shall constitute a waiver by customers for any and all such claims.

Certified by: [signature]

Quality Assurance Department

Dec/17/2018

NovoPro Biosciences Inc. VERIFIED Quality Control

278

NovoPro

For enjoyable discovery

Sample Information

Order ID	:GT31707-10
Name	:278
Sequence	:RWRWPIRRPPIRPPFYR
Lot. No	:GT31707-10-1207
Pump A	:0.1%Trifluoroacetic in 100% water
Pump B	:0.1%Trifluoroacetic in 100% acetonitrile
Total Flow	:1ml/min
Wavelength	:214nm
Analytial column type	:SHIMADZU Inertsil ODS-SP(4.6*250mm*5um)
Dissolution method	:100%H2O
Inj. Volume	: 15ul

Time	Module	Action	Value
0.01	Pumps	B.Conc	2
30.00	Pumps	B.Conc	65
33.00	Pumps	B.Conc	100
38.00	Pumps	B.Conc	100
40.00	Pumps	B.Conc	2
50.00	Controller	Stop	

Chromatogram

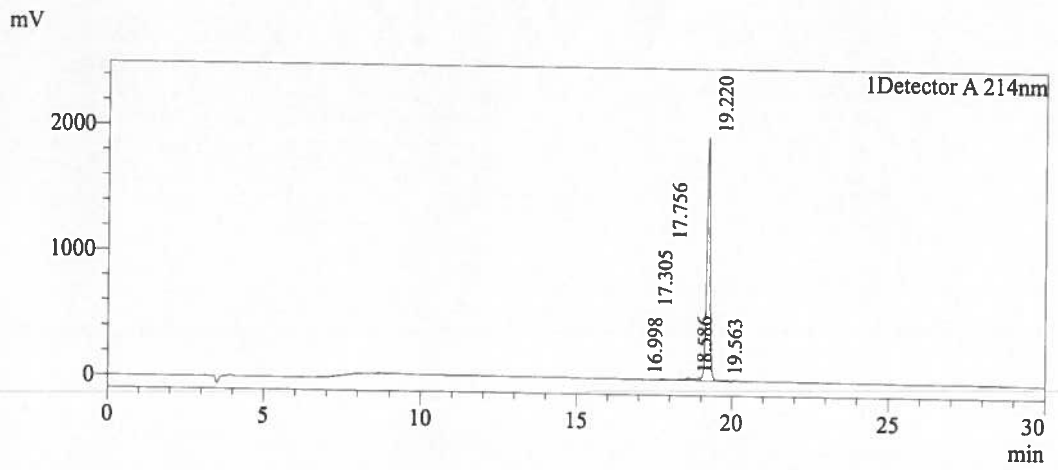

Peak Table

Detector A 214nm

Peak#	Ret. Time	Area	Height	Area%
1	16.998	14963	1564	0.100
2	17.305	4697	835	0.032
3	17.756	80394	13432	0.540
4	18.586	449919	17539	3.020
5	19.220	14186680	1933942	95.227
6	19.563	161057	4050	1.081
Total		14897709	1971362	100.000

278

MS Spectrum

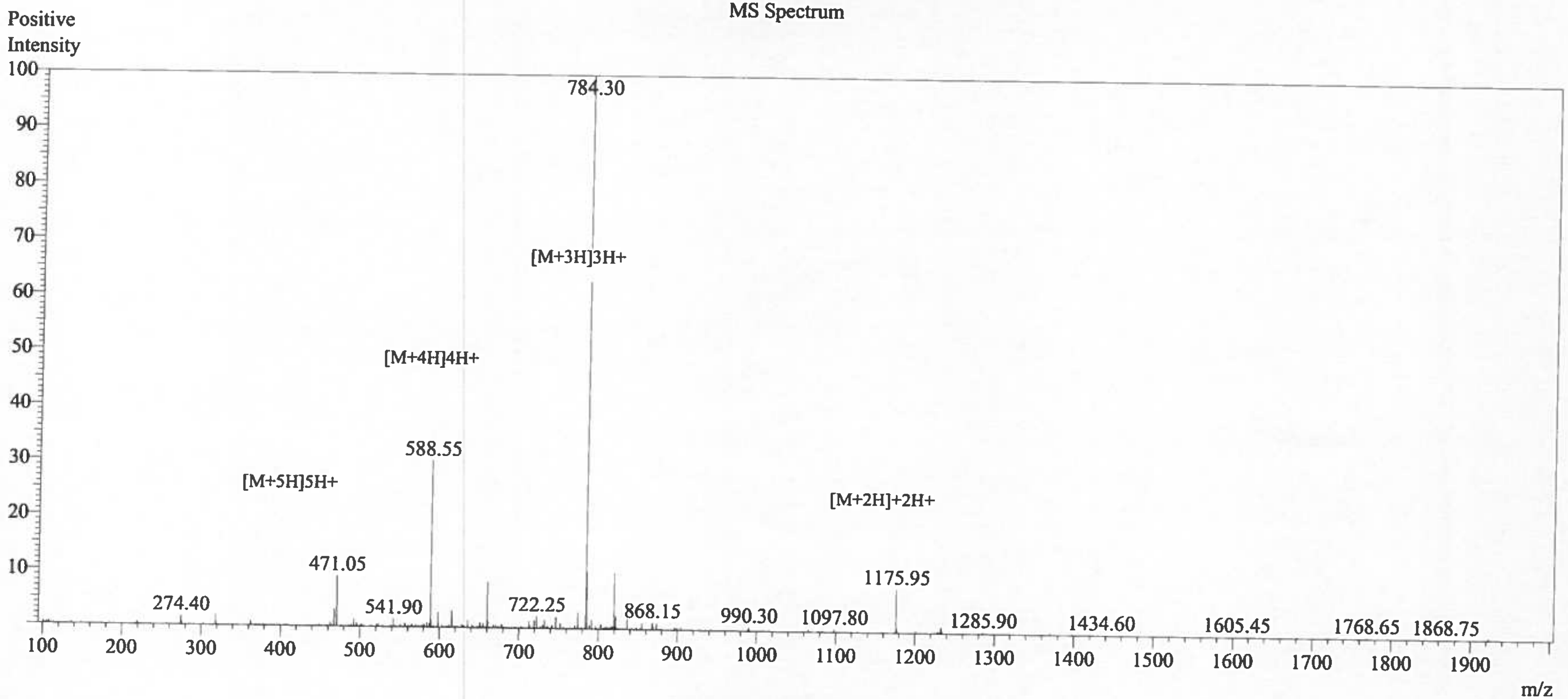

Sample Information

Dissolution method	:5%HAC+8%ACN+87%H2O	Interface	:ESI	Prerod Bias	:+4.5kv
Date Acquired	:2018/12/10 10:09:51	Nebulizing Gas Flow	:1.50L/min	Detector	:-0.2kv
Injection Volume	:0.2ul	CDL Temp	:250C	T.Flow	:0.2ml/min
Block Temp	:200	CDL Volt	:0v	B.conc	:50%H2O/50%MEOH

Order ID	:GT31707-10
Name	:278
Sequence	:RWRWPIRRPPIRPPFYR
Lot.No	:GT31707-10-1207
Theoretical	:2349.79
Observed	:2349.90

281

NovoPro

For enjoyable discovery

CERTIFICATE OF ANALYSIS

**Product Name**	GT31707-11
**Lot No.**	GT31707-11-1207
**Sequence**	RWRRPIRRRPIRPPFYW
**Dissolution condition**	100%H2O
**Length**	17AA
**Modification**	N/A
**Molecular Weight (MW)**	2408.86
**Storage**	-20°C

**Test Items**	**Specifications**	**Results**
**MW by MS**	2409.00	Conforms
**Purity by HPLC**	>95%	98.278%
**Peptide Content**	N/A	N/A
**Moisture content**	N/A	N/A
**Acetic acid content**	N/A	N/A
**Appearance**	White to off-white lyophilized powder	Conforms
**Quantity**	10mg	10.0mg

NovoPro Bioscience Inc. (hereafter NovoPro) warrants material of said quality at the time of sale. It is the sole responsibility of the customers to determine the adequacy of all materials for any intended or specific purpose or use. NovoPro's sole obligation is to replace the material up to the extent of the purchase price. This warranty applies only to products in original packaging and does not apply to a product which has been tampered with or altered in any way in or which has been misused or damaged by accident or negligence. All claims must be received writing (by fax or email) within 30 days from date when product arrive at the destination city and failure to do so shall constitute a waiver by customers for any and all such claims.

Certified by: [illegible]

Quality Assurance Department

Dec/18/2018

NovoPro Biosciences Inc. VERIFIED Quality Control

NovoPro

For enjoyable discovery

Sample Information

Order ID	:GT31707-11		
Name	:281		
Sequence	:RWRRPIRRRPIRPPFYW		
Lot. No	:GT31707-11-1207		
Pump A	:0.1%Trifluoroacetic in 100% water		
Pump B	:0.1%Trifluoroacetic in 100% acetonitrile		
Total Flow	:1ml/min		
Wavelength	:214nm		
Analytial column type	:SHIMADZU Inertsil ODS-SP(4.6*250mm*5um)		
Dissolution method	:100%H2O		
Inj. Volume	: 10ul		
Time	Module	Action	Value
0.01	Pumps	B.Conc	2
30.00	Pumps	B.Conc	65
33.00	Pumps	B.Conc	100
38.00	Pumps	B.Conc	100
40.00	Pumps	B.Conc	2
50.00	Controller	Stop	

Chromatogram

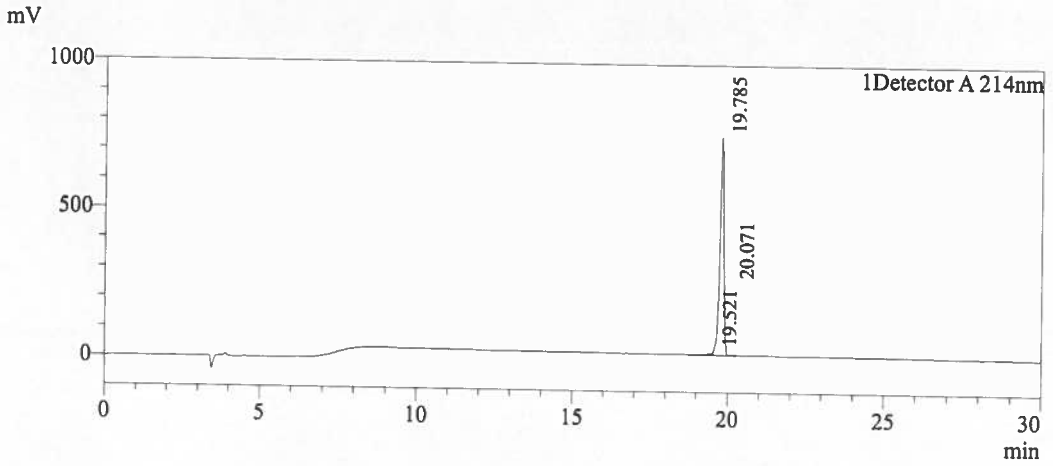

Peak Table

Detector A 214nm

Peak#	Ret. Time	Area	Height	Area%
1	19.521	104107	7240	1.593
2	19.785	6422006	731464	98.278
3	20.071	8407	829	0.129
Total		6534521	739534	100.000

281

MS Spectrum

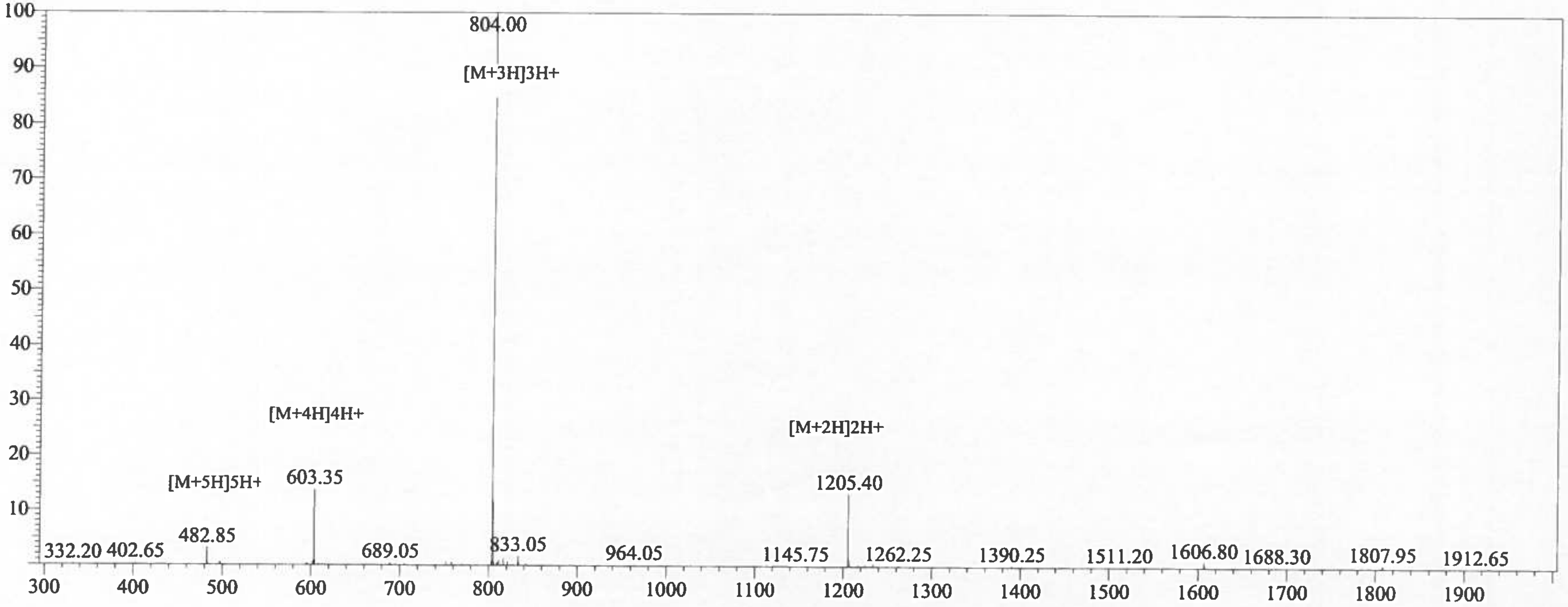

Sample Information

Dissolution method	:5%HAC+8%ACN+87%H2O	Interface	:ESI	Prerod Bias	:+4.5kv
Date Acquired	:2018/12/18 09:38:33	Nebulizing Gas Flow	:1.50L/min	Detector	:-0.2kv
Injection Volume	:0.2ul	CDL Temp	:250C	T.Flow	:0.2ml/min
Block Temp	:200	CDL Volt	:0v	B.conc	:50%H2O/50%MEOH

Order ID	:GT31707-11
Name	:281
Sequence	:RWRRPIRRRPIRPPFYW
Lot.No	:GT31707-11-1207
Theoretical	:2408.86
Observed	:2409.00

NovoPro

For enjoyable discovery

CERTIFICATE OF ANALYSIS

**Product Name**	GT31707-12
**Lot No.**	GT31707-12-1207
**Sequence**	RWRRPIRRRPIRPPFWR
**Dissolution condition**	100%H2O
**Length**	17AA
**Modification**	N/A
**Molecular Weight (MW)**	2401.87
**Storage**	-20°C

**Test Items**	**Specifications**	**Results**
**MW by MS**	2401.95	Conforms
**Purity by HPLC**	>95%	98.351%
**Peptide Content**	N/A	N/A
**Moisture content**	N/A	N/A
**Acetic acid content**	N/A	N/A
**Appearance**	White to off-white lyophilized powder	Conforms
**Quantity**	10mg	10.0mg

NovoPro Bioscience Inc. (hereafter NovoPro) warrants material of said quality at the time of sale. It is the sole responsibility of the customers to determine the adequacy of all materials for any intended or specific purpose or use. NovoPro's sole obligation is to replace the material up to the extent of the purchase price. This warranty applies only to products in original packaging and does not apply to a product which has been tampered with or altered in any way in or which has been misused or damaged by accident or negligence. All claims must be received writing (by fax or email) within 30 days from date when product arrive at the destination city and failure to do so shall constitute a waiver by customers for any and all such claims.

Certified by: 张成龙

Quality Assurance Department

Dec/19/2018

NovoPro Biosciences Inc. VERIFIED Quality Control

291

NovoPro

For enjoyable discovery

Sample Information

Order ID	:GT31707-12
Name	:291
Sequence	:RWRRPIRRRPIRPPFWR
Lot. No	:GT31707-12-1207
Pump A	:0.1%Trifluoroacetic in 100% water
Pump B	:0.1%Trifluoroacetic in 100% acetonitrile
Total Flow	:1ml/min
Wavelength	:214nm
Analytial column type	:SHIMADZU Inertsil ODS-SP(4.6*250mm*5um)
Dissolution method	:100%H2O
Inj. Volume	: 20ul

Time	Module	Action	Value
0.01	Pumps	B.Conc	2
30.00	Pumps	B.Conc	65
33.00	Pumps	B.Conc	100
38.00	Pumps	B.Conc	100
40.00	Pumps	B.Conc	2
50.00	Controller	Stop	

Chromatogram

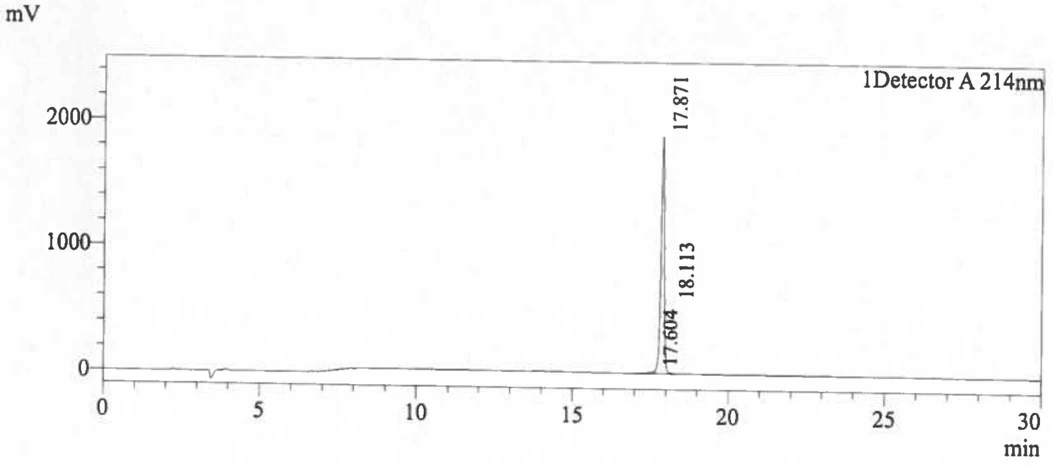

Peak Table

Detector A 214nm

Peak#	Ret. Time	Area	Height	Area%
1	17.604	199880	13540	1.378
2	17.871	14268508	1884504	98.351
3	18.113	39291	7128	0.271
Total		14507680	1905173	100.000

NovoPro

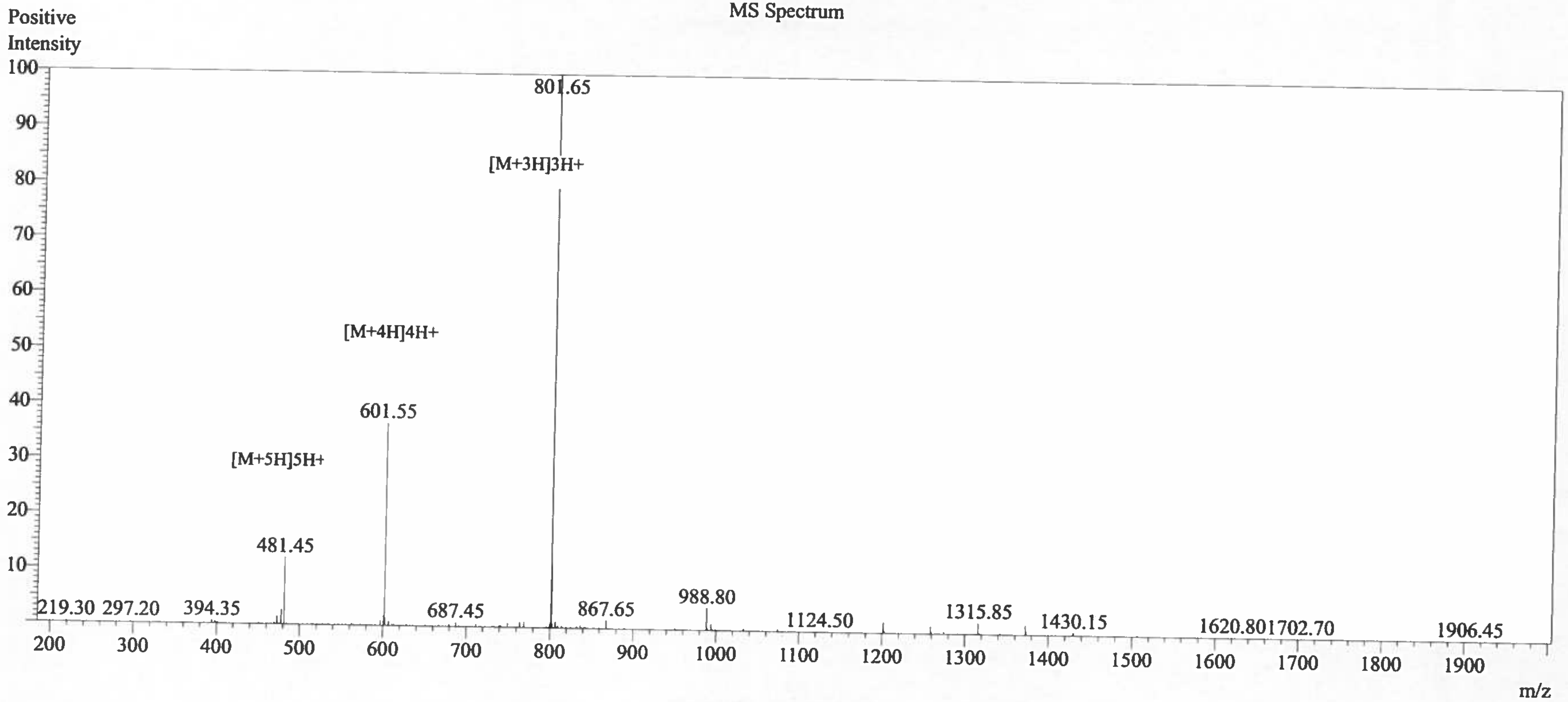

Sample Information

Dissolution method	:5%HAC+8%ACN+87%H2O	Interface	:ESI	Prerod Bias	:+4.5kv
Date Acquired	:2018/12/17 10:31:52	Nebulizing Gas Flow	:1.50L/min	Detector	:-0.2kv
Injection Volume	:0.2ul	CDL Temp	:250C	T.Flow	:0.2ml/min
Block Temp	:200	CDL Volt	:0v	B.conc	:50%H2O/50%MEOH

Order ID	:GT31707-12
Name	:291
Sequence	:RWRRPIRRRPIRPPFWR
Lot.No	:GT31707-12-1207
Theoretical	:2401.87
Observed	:2401.95

291
